# Long Non-coding RNA MEG3 Attenuates the Angiotensin II-Induced Injury of Human Umbilical Vein Endothelial Cells by Interacting With p53

**DOI:** 10.3389/fgene.2019.00078

**Published:** 2019-02-19

**Authors:** Jingwen Song, Songqun Huang, Kaizhong Wang, Wei Li, Lizhi Pao, Feng Chen, Xianxian Zhao

**Affiliations:** ^1^Department of Cardiovascularology, Shanghai Changhai Hospital, Second Military Medical University, Shanghai, China; ^2^Institute of Tumor, Second Military Medical University, Shanghai, China

**Keywords:** cardiovascular disease, HUVECs, Ang II, Meg3, p53

## Abstract

Angiotensin II (Ang II)-induced damage to endothelial cells (ECs) plays a crucial role in the pathogenesis of cardiovascular disease. This study aimed to investigate the role of maternally expressed gene 3 (Meg3) in endothelial cell injury. A lncRNA human gene expression microarray analysis was used to identify differentially expressed lncRNAs in human umbilical vein endothelial cell (HUVECs). Cell viability, apoptosis, and migration were then assessed Ang II-treated HUVECs. qRT-PCR and western blotting were performed to detect the expression level of p53 after Meg3 knockdown and overexpression. We observed that Ang II treatment decreased the Meg3 level in HUVECs. Next, both knockdown of Meg3 and Ang II decreased cell viability, increased apoptotic cell rate and impair migration function in HUVECs. Furthermore, overexpression of Meg3 inhibited cell apoptosis, and increased cell migration by enhancing p53 transcription on its target genes, including CRP, ICAM-1, VEGF, and HIF-1α. Our findings indicate that Meg3 might be associated with cardiovascular disease development.

## Introduction

Endothelial cells (ECs) are very commonly distributed in heart and other organs, which guard relevant tissues. In addition, ECs were found to be significant in pathological and physiological processes, such as atherosclerosis, inflammation, etc. (Borgo et al., 2016). Angiotensin II (Ang II) is an important peptide in renin-angiotensin system (RAS), and has been found to play a significant role in cardiovascular system (Wang et al., 2013). Previous studies have revealed Ang II-induced endothelial dysfunction has been used in various cardiovascular models, such as hypertension, and myocardial infarction (Dimmeler et al., 1997). Numerous factors, including reactive oxygen species (ROS) release, DNA damage, and inflammation, were considered to be associated with Ang II-induced endothelial apoptosis (Paravicini and Touyz, 2006; Caporali and Emanueli, 2011; Mendell and Olson, 2012; Wang et al., 2012), however, the mechanisms underlying Ang II-induced EC apoptosis remains poorly understood.

Long non-coding RNAs (lncRNAs) are very special transcripts, with non-protein coding property and more than 200 nucleotides. LncRNA has been considered as an important constituent of mammalian transcriptomes (about 4–9%) (Banfai et al., 2012; Takahashi and Carninci, 2014). Lots of studies have revealed lncRNAs have been involved in gene expression regulation and biological processes, such as epigenetics, and cell growth (Geisler and Coller, 2013; Karapetyan et al., 2013; Bonasio and Shiekhattar, 2014; Wang et al., 2014). Previous research has suggested that lncRNAs were associated with the cardiovascular diseases since lncRNAs were found in cardiovascular endothelial cells (Luther et al., 2005; Visel et al., 2010; Zhao et al., 2010), however, the function of lncRNAs in cardiovascular system needs further data to confirm.

Maternally expressed gene 3 (Meg3), an lncRNA, is widely expressed in many normal tissues. Previously studies have suggested that Meg3 is a tumor suppressor lncRNA, and Meg3 is down regulated in a number of different cancers such as breast, bladder and hepatocellular carcinoma and that its downregulation increases cell proliferation in those types of cancer (Zhang et al., 2003; Anwar et al., 2012; Ying et al., 2013; Sun et al., 2016). It is part of the DLK1-Meg3 locus located on human chromosome 14q32 (Miyoshi et al., 2000) and was believed to be associated with ECs’ injury (Zhang et al., 2003). Its expression level was also found to be related to downregulation in isoprenaline treated HUVECs (Yan Y.Y. et al., 2016). However, the role of Meg3 in HUVECs’ injury remains unclear.

In this study, we examined the roles of Meg3 in HUVECs and its potential mechanism. It was revealed that Meg3 expression level is reduced in HUVECs following treatment with Ang II, and subsequent bioinformatics analysis suggested that p53 may be a downstream target of Meg3.

## Materials and Methods

### Animals

C57BL/6 mice were obtained from Shanghai Slac Laboratory Animal Co. Ltd. (SCXK (Hu) 2018-0015). All animals were housed in a pathogen-free room with a controlled ambient temperature (23 ± 2°C) and humidity (55 ± 5%), and performed in accordance with the Guide for the Animal Research Committee of Changhai Hospital (Shagnhai, China).

### Animal Models and Treatment

Ang II was dissolved in 0.9% NaCl, and subcutaneously infused (1.4 mg /kg per day) for 4 weeks using an osmotic minipump (Alzet model 2004, Alza Corp) to obtain Ang II-induced cardiac hypertrophy. Saline-infused animals were used as controls with same procedure of Ang II-induced cardiac hypertrophy, except for the Ang II uses.

### Echocardiographic Measurements

Echocardiography was recorded based on previous study (Wu et al., 2018). Briefly, 1.5–2.5% isoflurane was continuously inhaled using Pour Fill R500IP (RWD lifescience, China). M-mode tracings derived from the short axis of the LV at the level of the papillary muscles were recorded. The left ventricular (LV) end-diastolic dimension (LVEDd) and LV end-systolic dimension (LVESd) were measured at the largest and smallest LV areas, respectively.

### Histological Analysis

Hearts were sectioned and stained with hematoxylin-eosin staining (HE). The cross-sectional areas of myocytes and fibrotic areas were measured using a digital image analysis system (Image-Pro Plus, version 6.0) from images captured from HE-stained sections.

### Cell Culture and Ang II Treatment

Human umbilical vein endothelial cells (HUVECs) were obtained from the Cell bank of Chinese Academy of Sciences (Shanghai, China) and were grown in Dulbecco’s modified Eagle’s medium (ScienCell Co., United States) that was supplemented with 10% fetal bovine serum (FCS) (Gibco Co., United States) in a humidified incubator with 5% CO_2_ at 37°C. HUVECs at passages 3–10 are used in the present study. The cells were treated with various concentrations (0.1–100 μM) of Ang II (Sigma-Aldrich, United States) for different incubation times (24, 48, 72 h).

### RNA Extraction and Quantitative Reverse Transcription PCR

Total RNA was harvested from HUVEC using TRIzol reagent (Invitrogen, Carlsbad, CA, United States) and the RNeasy Kit (Qiagen Co., Hilden, Germany) according to the manufacturer’s instructions, including a DNase digest ion step. Total RNA from each cell line was quantified using a NanoDrop ND-2000 (OD 260 nm, NanoDrop, Wilmington, DE, United States) RNA integrity was assessed using standard denaturing agarose gel electrophoresis, and the purity was judged by the ratio of absorbance at 260 nm to 280 nm (A260/A280). Reverse transcription was performed using 0.55 μg reverse transcription primes Odigo(dT) and 1 μg total RNA using K1622 revert aid first strand cDNA synthesis kit (Thermo Scientific Fermentas, United States). Quantitative real-time PCR (qRT-PCR) was performed using SYBR Premix EX TaqTM (TaKaRa, Dalian, China). Primers of Meg3 and other genes as well a hACTB as control were presented in Supplementary Table S2. The expression level of each gene was represented as the fold change using the 2^-ΔΔCt^ methods.

### Plasmid Construction and Transfection

The pcDNA-si-MEG3, pcDNA-MEG3-OE, pcDNA-si-p53, and their respective control vector were purchased from Guangzhou RiboBio Co., China. Cell transfection was performed with POLO3000 (Shanghai Ruisai Biotech Co., China) according to the manufacturer’s instructions.

### High Throughput lncRNA Expression Profile Analysis

The Affymetrix Human OE lncRNA (OE Biotech. Co., Shanghai, China) was used. Briefly, total RNAs were transcribed to double strand cDNAs and then synthesized cRNAs. Then, the 2nd cycle cDNAs were synthesized from cRNAs. Followed fragmentation and biotin labeling, the 2nd cycle cDNAs were hybridized onto the microarray. After washing and staining, the arrays were scanned by the Affymetrix Scanner 3000 (Affymetrix). The differential LncRNAs were screened out by using the following parameters: *p* < 0.05 and fold > 1.5. Hierarchical clustering analysis was employed on differentially expressed lncRNAs. The raw data of the microarray have been uploaded to GEO with the series record GSE123679.

### Western Blotting

Cells were lysed using lysis buffer supplemented with phenylmethylsulfonyl fluoride (1 mM) on ice. Then, the protein lysates were electrophoresed by using 10% SDS polyacrylamide gels and transferred to a PVDF membrane (Millipore, Billerica, MA, United States). Membranes were blocked in 5% non-fat milk solution at room temperature for 1 h and then incubated with the primary antibodies at 4°C overnight. Next, membranes were incubated with secondary antibodies labeled with HRP for 1 h at room temperature after three 5 min washes in triethanolamine buffered saline solution with Tween (TBS-T). Finally, the signals were detected by using an ECL kit (Pierce Biotech., Rockford, IL, United States) and the membranes were scanned and analyzed using Odyssey LICOR CLX infrared imaging system (Gene Co., United States). Tubulin was used as an internal control. The antibodies used for western blotting are listed in Supplementary Table S3.

### Transwell Migration Assay

Cell migration was determined using a transwell chamber (8 × 8 μm pore size). After digestion, a total of 4 × 10^3^ cells in 100 μL serum-free medium were plated to the upper chambers and 600 μL of medium containing 10% serum was used as a chemoattractant in the lower chambers. After 5 h, the cells on the upper side of the membrane were removed using cotton swabs, and the invaded cells on the lower side of the membrane were fixed, stained with GENMED crystal violet, and counted by using an using an GENMED crystal violet staining and inverted microscope at 100× magnification. The OD value was determined using a microplate reader at 570 nm.

### Apoptosis Analysis

Apoptosis was analyzed by flow cytometry using the Annexin V-PI detection kit. After transfection, cells were harvested for Annexin V-PI staining according to the manufacturer’s instructions (Tianjin Sungene Biotech Co., China). The apoptosis rate was calculated by flow cytometric data and the cell counts.

### Data Preprocessing

Unpaired *t*-tests were conducted applying GraphPad Prism version 6.00 for Windows, (GraphPad Software, La Jolla, CA, United States) ^1^. Asterisks in figures summarize *P*-values (^∗∗^*P* < 0.01; ^∗^*P* < 0.05).

## Results

### Meg3 Is Downregulated in HUVECs and Mice Treated With Ang II

**FIGURE 1:**
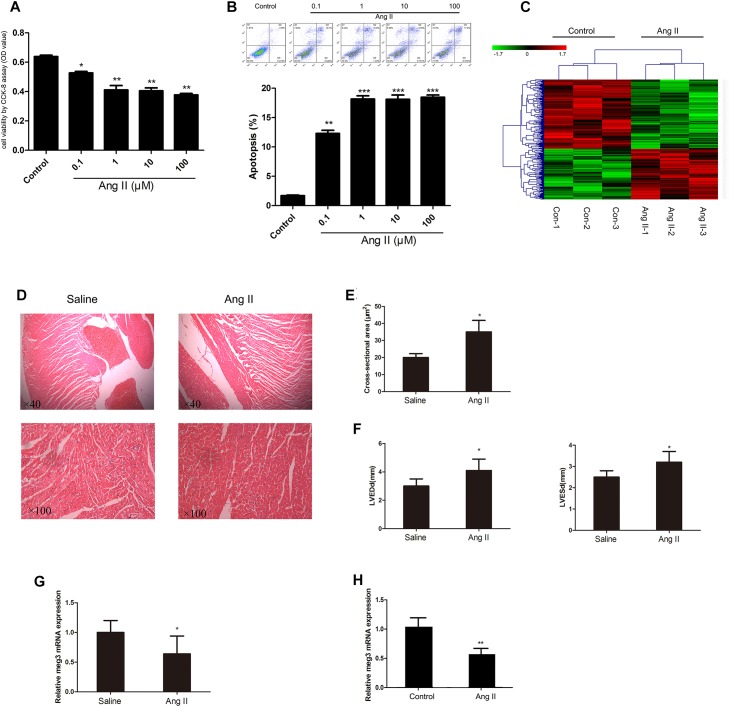
Effect of angiotensin II (Ang II) on human umbilical vein endothelial cells (HUVECs). **(A)** HUVECs were treated with Ang II at different concentrations (1, 10, and 100 μM) (*n* = 3/group). **(B)** Ang II-induced apoptosis of HUVECs (*n* = 3/group). **(C)** LncRNA expression profiles were generated from two groups of HUVECs (*n* = 3/group). **(D)** Histological analysis of heart slices by HE staining to assess cardiomyocyte cross-sectional areas in the hypertrophy and saline groups (*n* = 6 mice per group magnification). **(E)** Statistical results for the cell cross-sectional area in hypertrophy and saline groups (*n* > 100 cells per group). **(F)** Echocardiographic measurements of left ventricle end-diastolic dimension (LVEDd) and LV end-systolic dimension (LVESd) in hypertrophy and saline groups (*n* = 6 mice/group). **(G)** The expression level of Meg3 in hypertrophy and saline groups (*n* = 6 mice/group). **(H)** The expression level of Meg3 in two groups of HUVECs (*n* = 3/group). The data are shown as the mean ± SD, Student’s *t*-test, ^∗^*P* < 0.05; ^∗∗^*P* < 0.01 vs. control group.

We first investigated the effect of Ang II treatment on the cell viability by CCK-8 assay. Ang II decreased cell viability in a concentration-dependent manner (Figure 1A). Compared with the control, Ang II, at concentrations of 1, 10, and 100 μM, significantly reduced HUVECs’ viability (*P* < 0.01). Moreover, Annexin V-FITC/PI data revealed that HUVECs incubation with Ang II for 48 h resulted in apoptotic rate to 18.16 ± 0.95%, 18.11 ± 1.27%, and 18.45 ± 0.69%, for concentrations of 1, 10, and 100 μM, respectively (Figure 1B). In addition, the incubation time of Ang II, including 24, 48, and 72 h, was also investigated in our research (Supplementary Figure S1). Both 48 and 72 h showed significant cell viability reduction (*P* < 0.01), compared with the control group. Thus, in the following experiment, we incubated the HUVECs with Ang II of 1 μM for 48 h. To identify potential molecular factors associated with Ang II induced injury in HUVECs, we used Affy lncRNA gene expression microarray (Affymetrix Company, United States) to analyze differentially expressed lncRNAs in both normal and Ang II induced HUVECs. Then differentially expressed probes were calculated and 92728 genes were identified. As shown in Figure 1C, hierarchical clustering analysis showed the differential expression of 293 non-coding RNA transcripts (123 up- and 170 downregulated), with the *P* < 0.05 and absolute fold change (FC) > 1.5 (Supplementary Table S1). Among the differential lncRNAs, Meg 3 was decreased over 2.5-fold in the Ang II induced group compared to the control group. In addition, 4 weeks of Ang II infusion in mice led to significantly increased cell cross-sectional area (csa), left ventricle end-diastolic dimension (LVEDd), and LV end-systolic dimension (LVESd) (Figure 1D–F). Consistently, qRT-PCR analysis showed that Meg 3 was significantly down-expressed in Ang II induced group compared to control group (Figure 1G). Also, Meg 3 was significantly down-expressed in Ang II infusion group compared with saline controls (Figure 1H).

### Knockdown of Meg3 Reduces Viability, Promotes Apoptosis, and Impairs Migration in HUVECs

**FIGURE 2:**
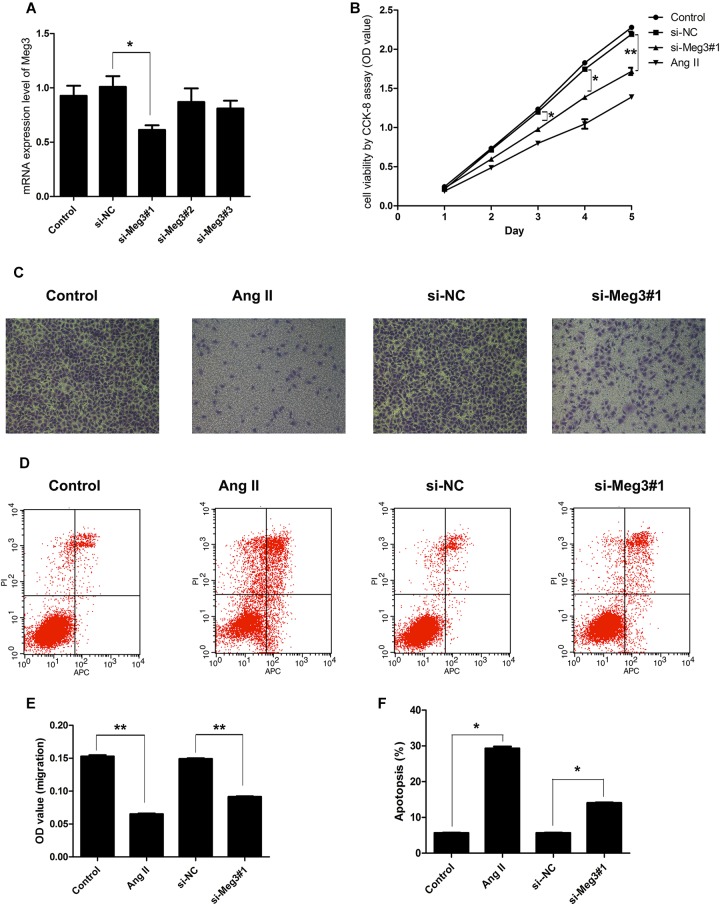
Suppression of Meg3 inhibited cell viability, migration, and promoted apoptosis in HUVECs. **(A)** HUVECs were transfected with three different sequences of Meg3 si-RNA (si-MEG1#1, si-MEG1#2, and si-MEG1#3), the expression of Meg3 in the transfected cells were then measured by qRT-PCR (*n* = 3/group). **(B)** Cell viability was determined by CCK-8 (*n* = 3/group). **(C)** Transwell assays on Meg3-silenced and Ang II-induced HUVECs (*n* = 3/group). **(D)** Apoptosis assay on Meg3-silenced and Ang II-induced HUVECs (*n* = 3/group). **(E)** Quantitative analysis of migration in Ang II-induced HUVECs (*n* = 3/group). **(F)** Quantitative analysis of the percentage of apoptotic cells in Ang II-induced HUVECs (*n* = 3/group). The data are shown as the mean ± SD, Student’s *t*-test, ^∗^*P* < 0.05; ^∗∗^*P* < 0.01.

To explore the functional effects of Meg3 on HUVECs, the expressions of Mg3 in HUVECs were altered by transfection with three different sequences of Meg3 siRNA (si-Meg3#1, si-Meg3#2, and si-Meg3#3). After transfection, the expression of Meg3 in cells was measured by QRT-PCR, and we found that both si-MEG3#1 significantly reduced Meg3 expression when compared to the si-NC group (*P* < 0.01, Figure 2A). Thus si-Meg3#1 transfected cells were used as a test group for the following experiments.

Next, we detected the impacts of Meg3 silence on HUVECs viability, migration, and apoptosis. As shown in Figure 2B, the cell viability was inhibited from days 3 to 5 by both Ang II and si-Meg3, which suggested that Ang II may inhibit HUVECs growth through Meg3 suppression. This hypothesis was further supported by HUVECs migration, and apoptosis induced by Ang II and Meg3 silence. Both Ang II and Meg3 silence could inhibit migration (Figure 2C) and increase apoptotic cell rate (Figure 2D), with significant differences compared to control group, respectively (*P* < 0.05, *P* < 0.01) (Figure 2E,F). These data indicated Ang II may inhibit HUVECs growth and migration, and increase apoptotic cell rate via downregulated Meg3 expression.

### Overexpressed Meg3 Increases Viability, Inhibits Apoptosis, and Enhances Migration Function in HUVECs Treated With Ang II

**FIGURE 3:**
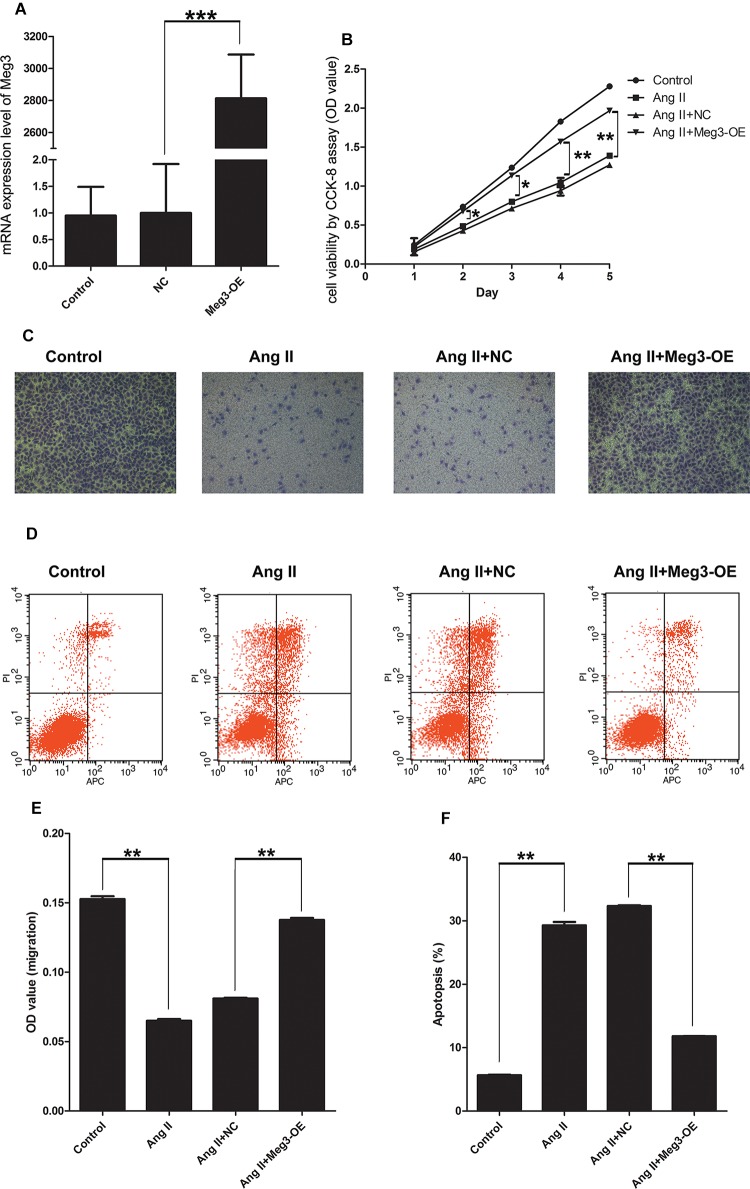
Meg3 overexpression suppresses cell viability, migration, and promoted apoptosis in HUVECs treated with Ang II. **(A)** qRT-PCR analysis of the expression of Meg3 in control or Meg3 stably transfected HUVECs (*n* = 3/group). **(B)** Cell viability was determined by CCK-8 (*n* = 3/group). **(C)** Transwell assay evaluates the effect of Meg3 overexpression on Ang II treated HUVECs (*n* = 3/group). **(D)** Apoptosis of HUVECs was determined by Annexin V/PI staining followed by flow cytometric analysis in HUVECs treated with Ang II (*n* = 3/group). **(E)** Quantitative analysis of migration in Ang II-induced HUVECs (*n* = 3/group). **(F)** Quantitative analysis of the percentage of apoptotic cells in Ang II-induced HUVECs (*n* = 3/group). The data are shown as the mean ± SD, Student’s *t*-test, ^∗^*P* < 0.05; ^∗∗^*P* < 0.01; ^∗∗∗^*P* < 0.001.

Since Meg3 was downregulated in HUVECs treated with Ang II, wegenerated Meg3-overexpressed HUVECs to observe its function. We generated Meg3-OE by stable transfection of lentivirus Meg3. QRT-PCR results confirmed that the expression level of Meg3 in Meg3-OE was upregulated by ∼2800-fold (Figure 3A). Compared with HUVECs transfected with non-specific scramble control (NC), CCK-8 assay showed that overexpression of Meg3 in HUVECs significantly increased the cell growth from days 2 to 5 treated with Ang II (Figure 3B). In addition, Annexin V-FITC/PI assay showed that overexpression of Meg3 could remarkably inhibit cell apoptosis (Figure 3D,F). Transwell assay showed that HUVECs migration was remarkably impaired in those transfected with Meg3 compared with those transfected with the control (Figure 3C,E). Taken together, these data suggested that overexpressed Meg3 could regulate HUVECs’ growth, apoptosis and migration treated with Ang II.

### Meg3 Regulates the p53 Pathway in HUVECs Activated by Ang II

**FIGURE 4:**
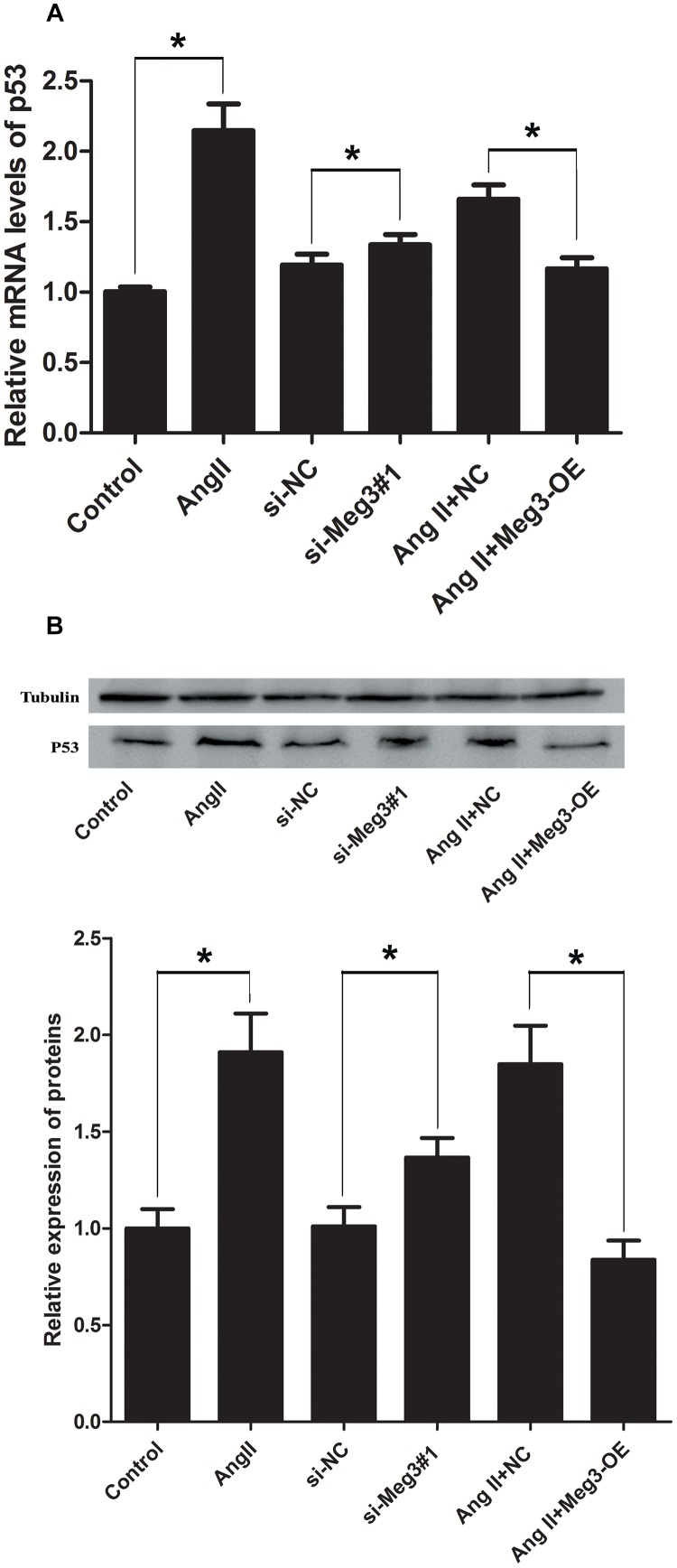
Ectopic expression of Meg3 regulates the level of p53 in HUVECs treated with Ang II. **(A)** qRT-PCR analysis of Meg3 after ectopic expression of Meg3 (*n* = 3/group). **(B)** Western blot analysis of Meg3 after ectopic expression of Meg3 (*n* = 3/group). The data are shown as the mean ± SD, Student’s *t*-test, ^∗^*P* < 0.05; ^∗∗^*P* < 0.01.

Previous studies have suggested that Meg3 functions through the activation of p53, leading to an increase in p53 protein level and stimulated p53-dependent transcription in a variety of cancer cells (Zhou et al., 2007; Lu et al., 2013; Hu et al., 2016; Li et al., 2016). The involvement of p53 in HUVECs was indicated by aforementioned Ang II induction, Meg 3 silence and Meg 3 overexpression. The relative expressions (2^-ΔΔCt^) of p53 mRNA in the Ang II, si-Meg3#1, and Ang II +NC groups were 2.15 ± 0.33, 1.34 ± 0.13, and 1.66 ± 0.17, respectively, which were significantly higher than the control, si-NC, and Ang II +Meg3-OE groups, respectively (*P* < 0.05, Figure 4A). Western blotting demonstrated that Ang II led to increased levels of p53 in HUVECs compared to the control group (Figure 4B). This increase was eliminated by the upregulated Meg3, which specifically targets Meg3 (Meg3-OE, Figure 4B). In addition, Meg3 silence also increased the level of p53 compared to the scrambled Meg3 siRNA (si-NC). These data indicate that Meg3 induces the upregulation of p53 and enhances its transcription activity in HUVECs.

**FIGURE 5:**
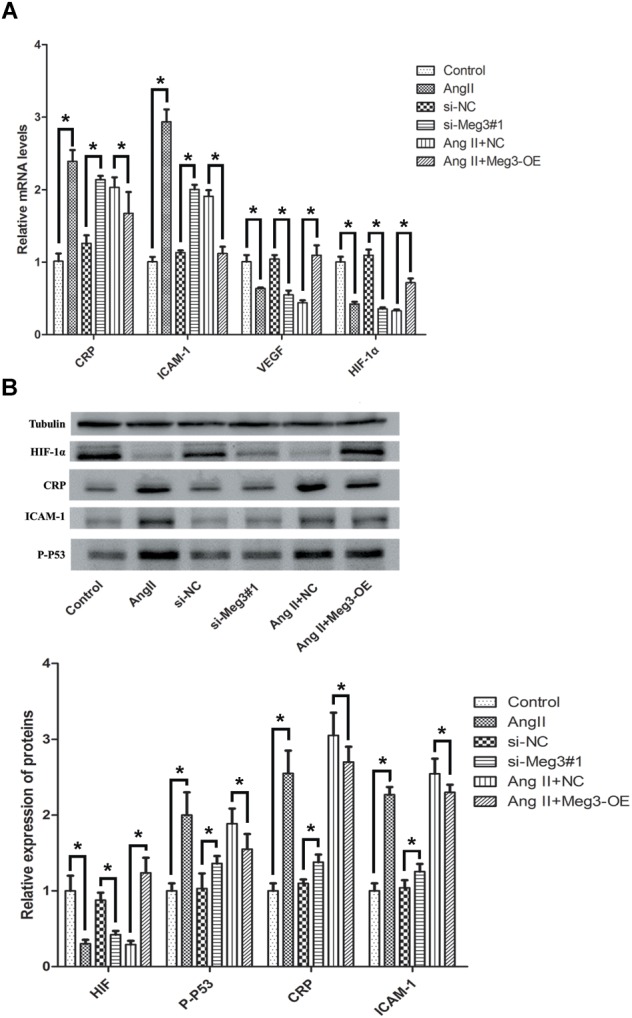
Ectopic expression of Meg3 regulates the downstream proteins level of p53 in HUVECs treated with Ang II. **(A)** qRT-PCR analysis of mRNA levels after ectopic expression of Meg3 (*n* = 3/group). **(B)** Western blot analysis of CRP, ICAM-1, p-p53, and HIF-1α after ectopic expression of Meg3 (*n* = 3/group). The data are shown as the mean ± SD, Student’s *t*-test, ^∗^*P* < 0.05.

By considering the association of Meg3 with p53’s activation, we next detected the effects of knockdown and overexpression of Meg3 on downstream gene expression of p53, including CRP, ICAM-1, VEGF, and HIF-1α. Interestingly, the mRNA expressions of CRP and ICAM-1 were upregulated after Meg3 silence, while VEGF, and HIF-1α were downregulated in the si-Meg3#1 group, compare to the scrambled Meg3 siRNA (si-NC) (Figure 5A). Ang II also has the same changes with the Meg3 silence in HUVECs. Western blotting demonstrated that both Ang II and si-Meg3 caused increase of CRP, ICAM-1, and p-p53 and reduction of HIF-1α. These protein changes were also eliminated by the overexpressed Meg3 (Meg3-OE, Figure 5B).

### Knockdown of Meg3 Regulates p53’s Transcriptional Activation

**FIGURE 6:**
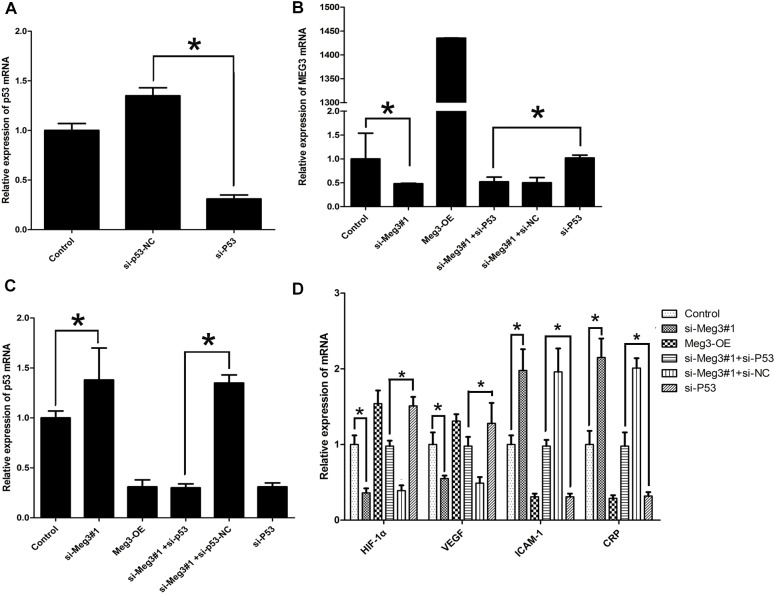
Ectopic expression of Meg3 regulates p53’s transcriptional activation. **(A)** The expressions of p53 in p53 silenced cells were then measured by qRT-PCR (*n* = 3/group). **(B)** The expression of Meg3 in the transfected cells were then measured by qRT-PCR (*n* = 3/group). **(C)** The expression of p53 in the transfected cells were then measured by qRT-PCR (*n* = 3/group). **(D)** The expression of p53’ downstream genes in the transfected cells were then measured by qRT-PCR (*n* = 3/group). The data are shown as the mean ± SD, Student’s *t*-test, ^∗^*P* < 0.05.

To validate the necessity of p53 in Meg3-mediated processes, si-p53 (p53 mutant lacking transcriptional activity) were constructed and confirmed by qRT-PCR (Figure 6A). As showed in Figure 6B, block of p53 (si-p53) had no effect on Meg3 expression, while Meg3 silence increased the expression of p53 (Figure 6C), suggesting that Meg3 was targeting p53. In consistent with previous result, the mRNA expressions of CRP and ICAM-1 were downregulated after p53 silence, while VEGF and HIF-1α were upregulated in the si-p53 group (Figure 6D). All these changes were also eliminated by the inhibition of Meg3. Thus, silence Meg3 could directly increase p53 expression.

## Discussion

Non-coding RNAs are a promising class of regulators. The distribution of lncRNA of varying size greater than 200 nt in tissues and cells plays an important role in regulating the growth and development of the organism. Extensive and systematic research confirmed that lncRNAs may be involved in multiple processes, including growth, development, metabolism, function, and apoptosis in cells (Manrique et al., 2009; Marampon et al., 2013; Cimen et al., 2016).

Ang II activates diverse signaling cascades by binding to the Ang II type 1 receptor, leading to ECs dysfunction, including apoptosis (Li et al., 2014), however, the mechanisms underlying this process are complex. The present study revealed the same phenomenon that Ang II produced an elevation in the rate of apoptosis of HUVECs. Of note, certain previous studies have investigated several lncRNAs have involved in the ECs’ function. Michalik et al. (2014) suggested that MALAT1 was significantly increased by hypoxia and silenced MALAT1 increased migration and inhibited proliferation of ECs. Furthermore, knockdown of MALAT1 *in vivo* inhibited proliferation of endothelial cells and reduced neonatal retina vascularization (Michalik et al., 2014). In addition, Yan Y.Y. et al. (2016) have found arrest-specific transcript 5 (GAS5) and Meg3 were significantly downregulated in isoprenaline treated HUVECs. In view of this, we directly screened normal and Ang II treated HUVECs and identified a significantly downregulated lncRNA, Meg3, in the Ang II (Figure 1 and Supplementary Table S1), which was confirmed by qRT-PCR analysis. In present study, we investigated the association between Meg3 and Ang II-induced HUVECs’ injury. We have reported, for the first time to our knowledge, the Meg3 expression was decreased following Ang II treatment. This reduction was accompanied by a decrease in cell viability and migration. Apoptosis of ECs has been considered a crucial progress of endothelial dysfunction, because endothelial cell apoptosis is an initial step and a well-acknowledged mechanism of microvascular obliteration (Wang et al., 2010). Considering of this, inhibition of endothelial cell apoptosis may be a therapeutic method to prevent cardiovascular diseases, including hypertension. In the present study, Ang II treatment significantly induced apoptosis, which is consistent with previous study (Liu et al., 2013). The mechanisms of action of Meg3 in HUVECs still remain controversial, some studies found that overexpression of Meg3 in endothelial cells caused a decrease in cell proliferation, while Meg3 knockdown in HUVECs significantly induced proliferation and inhibited apoptosis (He et al., 2017; Wu et al., 2017; Wang et al., 2018). In our research, Meg3 knockdown in HUVECs significantly induced apoptosis and inhibited proliferation, which is similar to previous study (Ruan et al., 2018). Thus, the role of Meg3 seems to be complex although in same cell line, and we need more time to figure out the mechanism of Meg3, and the cell growth condition maybe contribute to the different roles of Meg3.

We next explored the mechanism of Meg3 in Ang II treated HUVECs. P53 is well-known for mediating cell apoptosis, promoting non-apoptotic cell death, and programming necrosis (Sturm et al., 1999; Zhao et al., 2015; Yan H. et al., 2016). In our research, activated p53 is shown in Ang II treated and Meg3 silenced HUVECs, while overexpressed Meg3 was shown to attenuate or even reverse Ang II-induced upregulated p53 level. Therefore, it is likely that knockdown Meg3 activates p53 through inhibition of p53 ubiquitination and blockage of p53 degradation. Indeed, as the most intensive transcription factor, p53 tetramers bind to a variety of regulatory elements recruit multiple transcriptional co-regulatory factors, such as chromatin remodeling complexes, histone modification enzymes, and transcription. Knockdown Meg3 can activate p53’s transcriptional activity, which is active as a homotetramer whose tetramerization is pivotal for its function and plays a vital role in the regulation of p53 activity. However, previous study showed Meg3 knockdown in HUVECs significantly downregulated p53 expression under high glucose induction (Wang et al., 2018), which suggested p53 expression was partially involved in external environment, and the effects between external environment and Meg3 on p53 expression need more research to confirm.

Cell adhesion molecule-1 (CAM-1) is very common in human atherosclerotic plaques and can be regulated by various stimuli (Haverslag et al., 2008), suggesting that ICAM-1 plays a role in atherosclerosis. Previous study has revealed that ICAM-1 expressed in parallel with the p21, which is a target of p53, and induced by p53 in an NF-κB-independent manner in senescent human cells (Gorgoulis et al., 2005). High expressed serum C-reactive protein (CRP) has been considered as a sensitive cardiovascular risk factor (Liuzzo et al., 1994; Thompson et al., 1995; Koenig et al., 1999). Liang et al. (2006) elegantly presented data on the signaling pathway of CRP in ECs. Meanwhile, increasing numbers of criticisms have been raised on the use of commercial preparations of CRP concerning to the incompletely defined and biologically active contaminants. Our experimental data shows that in absence of proper p53 activity, silenced Meg3 plays auxiliary role in upregulating ICAM-1 and CRP levels in HUVECs.

Vascular endothelial growth factor (VEGF) plays an important role in mediating various response in the vascular endothelial cells, including migration, which is necessary for atherosclerosis and tumor angiogenesis (Ferrara et al., 2003; Soda et al., 2013; Bartolotti et al., 2014). Accordingly, depletion of VEGF by anti-VEGF treatment may induce compensatory physiological responses. VEGF and VEGFR levels following anti-VEGF treatment would be a key indicator of treatment outcome for intraocular neovascular diseases. Our results show that Meg3 down-regulation induced the down-regulated VEGF and HIF-1α expression. Meg3 overexpression rescued ANG-II-induced alteration in gene expression, confirming the important role of Meg3 in ANG II-mediated endothelial cell injury. Moreover, our data indicate that p53 is an important regulatory factor of Meg3 function. Compared with the control group, Meg3 knockdown induced p53 up-regulation, leading to the down-regulation of VEGF and HIF-1α and the up-regulation of CRP and ICAM-1. In contrast, Meg3 overexpression induced p53 down-regulation, leading to VEGF and HIF-1α up-regulation and CRP and ICAM-1 down-regulation. These results confirm that, in endothelial cells, Meg3 signaling is mediated by p53, leading to the regulation of p53-targeted genes, VEGF, HIF-1α, CRP, and ICAM-1.

## Conclusion

Our study confirms that the Meg3 is an important regulator of Ang II-induced endothelial cell injury and confirmed that Meg3 signaling is mediated by p53, leading to alteration of the expression of p53-related genes, VEGF, HIF-1α, CRP, and ICAM-1. Our findings provide novel insights on the role of Meg3 in endothelial cell injury and suggest Meg3 as a potential target to prevent and repair endothelial cell damage for the prevention and treatment of cardiovascular diseases.

## Author Contributions

JS, SH, and KW conducted the experiments and analyzed data. WL helped with the experiments and was a major contributor in writing the manuscript. LP helped with the experiments in the revision process, especially the *in vivo* experiments. FC and XZ conceived the design and supervised the research process. All authors read and approved the final manuscript.

## Conflict of Interest Statement

The authors declare that the research was conducted in the absence of any commercial or financial relationships that could be construed as a potential conflict of interest.

## Footnotes

1 www.graphpad.com

## Abbreviations

Ang II: angiotensin II
CAM-1: cell adhesion molecule-1
CRP: C-reactive protein
ECs: endothelial cells
HUVECs: human umbilical vein endothelial cells
lncRNAs: long non-coding RNAs
Meg3: maternally expressed gene 3
RAS: renin-angiotensin system
VEGF: vascular endothelial growth factor
