# Supplementary material for: Long Non-coding RNA MEG3 Attenuates the Angiotensin II-Induced Injury of Human Umbilical Vein Endothelial Cells by Interacting With p53
Source: Front Genet. 2019 Feb 19;10:78. doi: 10.3389/fgene.2019.00078 (PMC6389612; doi:10.3389/fgene.2019.00078)
Supplement: Supplementary file 1 [file Table_1.DOCX]

**Long Noncoding RNA MEG3 attenuates the angiotensin II-induced Injury of human umbilical vein endothelial cells by interacting with p53**

Songqun Huang^1,^ ^†^, Jingwen Song^1, †^, Kaizhong Wang^1^, Wei Li^2^, Lizhi Pao^1^, Feng Chen^1,^*, Xianxian Zhao^1,^*

^1^ Department of Cardiovascularology, Shanghai Changhai Hospital, Second Military Medical University, Shanghai, 200433, China.

^2^ Institute of tumor, Second Military Medical University, Shanghai, 200433, China.

**†The first three authors** contributed equally to this work.

***Correspondence:**

Feng Chen

Email: chenfeng2013@ 163.com

Xianxian Zhao

Email: xianxianzhao2017@163.com


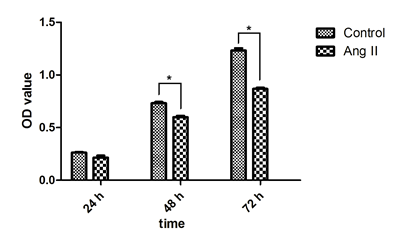


Figure S1. Cell viability was determined by CCK-8

Table S1. Probe sets (n=293) that are significantly differentially expressed between Ang II and controls.

| IProbe Set ID | lncRNA accession number inLNCipedia | p value | FC (abs) | Regulation | Control-1 | Control-2 | Control-3 | Ang II-1 | Ang II-2 | Ang II-3 |
| --- | --- | --- | --- | --- | --- | --- | --- | --- | --- | --- |
| TC0100000183.oe.1 | lnc-PGD-3 | 3.21E-04 | 1.601455 | down | 11.9666 | 12.0373 | 11.84419 | 11.2746 | 11.23902 | 11.29632 |
| TC0100002148.oe.1 | lnc-AL162431.1-1 | 9.11E-04 | 1.6212099 | down | 4.615182 | 4.599233 | 4.443468 | 3.81545 | 3.783297 | 3.967923 |
| TC0100002909.oe.1 | lnc-FAM138A-7 | 0.001812 | 1.6183103 | up | 3.299281 | 3.173684 | 3.264827 | 4.113149 | 3.844452 | 3.863656 |
| TC0100003474.oe.1 | lnc-KIAA0319L-3 | 0.010826 | 1.5940932 | up | 3.339426 | 3.14833 | 2.891879 | 3.657587 | 3.830474 | 3.909781 |
| TC0100003994.oe.1 | lnc-MCOLN3-1 | 0.044239 | 1.8212214 | up | 4.850457 | 4.934904 | 4.853509 | 5.974458 | 6.104335 | 5.154796 |
| TC0200000028.oe.1 | lnc-SNTG2-6 | 0.044944 | 1.5843456 | up | 14.69161 | 14.28326 | 14.8141 | 14.9472 | 15.32449 | 15.50894 |
| TC0200001714.oe.1 | lnc-CSRNP3-7 | 0.009227 | 1.5776614 | down | 3.230724 | 3.596128 | 3.39846 | 2.897181 | 2.77176 | 2.583007 |
| TC0200001952.oe.1 | lnc-GLS-2 | 0.033401 | 1.5206774 | up | 3.946819 | 3.644979 | 3.618552 | 4.034061 | 4.55885 | 4.431582 |
| TC0200002165.oe.1 | lnc-VWC2L-2 | 0.012686 | 1.6268038 | up | 3.882621 | 3.353684 | 3.640944 | 4.39031 | 4.380606 | 4.212454 |
| TC0200002357.oe.1 | lnc-SP140L-6 | 0.028769 | 1.5173801 | down | 11.01221 | 11.23788 | 10.62466 | 10.32887 | 10.39069 | 10.35044 |
| TC0200004006.oe.1 | lnc-MZT2A-1 | 0.00538 | 1.508201 | up | 2.413092 | 2.591549 | 2.250028 | 2.959029 | 2.975144 | 3.098982 |
| TC0200004028.oe.1 | lnc-LYPD1-1 | 0.02626 | 1.7391355 | down | 3.988856 | 3.292084 | 3.607985 | 2.605861 | 2.984272 | 2.903682 |
| TC0200004151.oe.1 | lnc-ARL5A-1 | 0.043546 | 1.5141052 | up | 4.975039 | 5.38413 | 5.111781 | 5.809987 | 6.012819 | 5.44354 |
| TC0300000249.oe.1 | lnc-TGFBR2-7 | 0.004314 | 1.5485531 | down | 4.664954 | 4.543963 | 4.409405 | 3.95096 | 4.019381 | 3.755219 |
| TC0300000335.oe.1 | lnc-EXOG-1 | 0.002256 | 1.6607176 | down | 8.782784 | 9.007427 | 9.136271 | 8.255837 | 8.203218 | 8.272008 |
| TC0300002706.oe.1 | lnc-NAA50-2 | 0.008436 | 1.6215397 | up | 4.729723 | 4.895976 | 4.871208 | 5.28593 | 5.552384 | 5.750687 |
| TC0300003358.oe.1 | lnc-MASP1-2 | 0.010572 | 1.5886453 | up | 14.07324 | 13.86875 | 14.27798 | 14.56804 | 14.79867 | 14.85665 |
| TC0400000473.oe.1 | lnc-KIT-1 | 0.005296 | 1.533492 | down | 3.374689 | 3.701877 | 3.672171 | 3.02959 | 2.891423 | 2.977263 |
| TC0400001097.oe.1 | lnc-HHIP-1 | 0.011219 | 1.5845734 | up | 2.749506 | 2.855338 | 2.832506 | 3.624011 | 3.185312 | 3.620311 |
| TC0400001162.oe.1 | lnc-ARFIP1-8 | 0.036858 | 1.5549248 | up | 3.366678 | 3.697087 | 3.105122 | 3.821706 | 4.035904 | 4.221812 |
| TC0400002198.oe.1 | lnc-ADH5-1 | 0.041903 | 1.5411688 | down | 6.851242 | 6.495242 | 7.068231 | 6.425175 | 6.133876 | 5.983588 |
| TC0400002220.oe.1 | lnc-PPP3CA-3 | 0.028513 | 1.7150835 | up | 2.900003 | 2.762748 | 3.045483 | 3.279403 | 4.025887 | 3.737782 |
| TC0500001508.oe.1 | lnc-PTTG1-11 | 5.78E-04 | 1.5836092 | down | 3.861579 | 3.701796 | 3.723308 | 3.086704 | 3.029315 | 3.181015 |
| TC0500002706.oe.1 | lnc-PGGT1B-5 | 0.04083 | 1.5800966 | down | 5.54127 | 5.620804 | 4.960511 | 4.844099 | 4.71846 | 4.579989 |
| TC0500003231.oe.1 | lnc-KCNMB1-7 | 0.001706 | 1.6497284 | up | 2.86517 | 2.751999 | 2.759058 | 3.341093 | 3.638756 | 3.563063 |
| TC0600000234.oe.1 | lnc-PPP1R3G-7 | 0.02802 | 1.5813003 | down | 7.484391 | 7.798818 | 7.20828 | 6.846612 | 6.998108 | 6.663437 |
| TC0600001171.oe.1 | lnc-POU3F2-5 | 0.032453 | 1.6193607 | down | 4.5212 | 4.7114 | 4.094848 | 3.514281 | 3.873336 | 3.853557 |
| TC0700000661.oe.1 | lnc-ZNF273-8 | 0.036244 | 1.6147949 | down | 7.601118 | 7.991068 | 7.474886 | 7.289253 | 6.966346 | 6.737419 |
| TC0700001778.oe.1 | lnc-THSD7A-4 | 0.041408 | 1.5218427 | down | 6.234688 | 6.781033 | 6.514679 | 6.050961 | 6.016999 | 5.644983 |
| TC0700002105.oe.1 | lnc-TNS3-1 | 0.047572 | 1.5378697 | up | 2.895294 | 3.114784 | 2.691501 | 3.841286 | 3.208651 | 3.514441 |
| TC0800000182.oe.1 | lnc-LPL-3 | 0.036935 | 1.5608357 | down | 7.711732 | 8.138466 | 7.69972 | 7.413814 | 7.294695 | 6.914451 |
| TC0800000732.oe.1 | lnc-HNF4G-6 | 0.016859 | 1.5546358 | down | 10.17016 | 10.71527 | 10.51363 | 9.854363 | 9.777281 | 9.857686 |
| TC0800000740.oe.1 | lnc-PKIA-1 | 6.80E-04 | 1.5068676 | down | 5.840969 | 5.673128 | 5.73708 | 5.170459 | 5.087405 | 5.218654 |
| TC0800000991.oe.1 | lnc-RP11-422N16.3.1-7 | 0.03942 | 1.5245966 | up | 5.858479 | 5.977794 | 5.696441 | 6.101261 | 6.727102 | 6.529634 |
| TC0800001448.oe.1 | lnc-FAM86B2-6 | 0.03242 | 1.829121 | down | 6.133097 | 6.587268 | 6.301739 | 5.465743 | 5.880705 | 5.062205 |
| TC0800001533.oe.1 | lnc-ENTPD4-2 | 0.023994 | 1.5440797 | up | 6.061362 | 5.949613 | 6.225722 | 6.836981 | 6.391457 | 6.8885 |
| TC0800002013.oe.1 | lnc-PAG1-11 | 0.010161 | 1.5101194 | down | 4.32393 | 4.573217 | 4.156386 | 3.83369 | 3.672661 | 3.763193 |
| TC0800002043.oe.1 | lnc-C8orf59-5 | 0.002487 | 1.5814064 | up | 3.056527 | 2.89821 | 3.006141 | 3.785546 | 3.490363 | 3.668593 |
| TC0800002118.oe.1 | lnc-RBM12B-4 | 0.034351 | 1.538019 | down | 7.078338 | 6.580526 | 6.452755 | 6.137626 | 6.124347 | 5.986425 |
| TC0800002166.oe.1 | lnc-RPL30-5 | 0.049337 | 1.6260418 | down | 4.43808 | 4.965512 | 4.188507 | 3.854341 | 3.995197 | 3.638467 |
| TC0900000447.oe.1 | lnc-FAM27C-5 | 0.003759 | 1.6216233 | down | 7.132744 | 7.066738 | 6.86884 | 6.488314 | 6.21152 | 6.276171 |
| TC0900001344.oe.1 | lnc-UAP1L1-3 | 0.03347 | 1.5125561 | down | 5.677912 | 5.846087 | 5.787944 | 5.329225 | 5.378982 | 4.812769 |
| TC0X00001147.oe.1 | lnc-RBBP7-3 | 0.016453 | 1.5547596 | down | 5.752944 | 5.797822 | 5.736444 | 5.272948 | 4.807857 | 5.296331 |
| TC0X00001427.oe.1 | lnc-SPIN4-2 | 0.00833 | 1.8373104 | down | 8.630256 | 9.116982 | 8.620749 | 7.820958 | 8.064107 | 7.850138 |
| TC0X00001543.oe.1 | lnc-BRWD3-1 | 0.012525 | 1.7540234 | down | 10.25278 | 10.61758 | 10.34268 | 9.88799 | 9.516819 | 9.37623 |
| TC0X00001681.oe.1 | lnc-IL13RA2-3 | 0.04523 | 1.6958792 | down | 7.175052 | 6.935412 | 6.855295 | 6.471671 | 6.475456 | 5.732531 |
| TC0X00001714.oe.1 | lnc-RNF113A-3 | 0.034925 | 1.5566267 | down | 11.32127 | 11.30293 | 10.77015 | 10.42221 | 10.67827 | 10.3786 |
| TC1000000507.oe.1 | lnc-PPYR1-2 | 0.002329 | 1.5503476 | down | 5.325214 | 5.630554 | 5.463758 | 4.890782 | 4.821991 | 4.808979 |
| TC1000000828.oe.1 | lnc-C10orf57-4 | 0.023469 | 1.5332628 | down | 3.876484 | 3.910978 | 4.060324 | 3.096973 | 3.25426 | 3.646738 |
| TC1000000964.oe.1 | lnc-HELLS-2 | 0.017048 | 1.5952493 | down | 7.771425 | 7.720715 | 7.759439 | 7.402311 | 7.002342 | 6.825581 |
| TC1000001195.oe.1 | lnc-VTI1A-1 | 0.014785 | 1.6104499 | up | 4.318686 | 3.953194 | 4.404496 | 5.016585 | 4.724645 | 4.997537 |
| TC1000001632.oe.1 | lnc-PTPLA-2 | 0.045437 | 1.5788126 | up | 3.401404 | 3.347638 | 3.958081 | 4.156391 | 4.063753 | 4.463498 |
| TC1000001644.oe.1 | lnc-C10orf113-3 | 0.006105 | 1.546095 | up | 3.896758 | 3.603558 | 3.743426 | 4.231483 | 4.519683 | 4.378464 |
| TC1000002650.oe.1 | lnc-EBF3-10 | 0.029065 | 1.6754324 | up | 6.75976 | 6.190869 | 6.229852 | 6.884962 | 7.29303 | 7.236089 |
| TC1100000357.oe.1 | lnc-FIBIN-2 | 0.010506 | 1.5122974 | down | 3.611165 | 3.380933 | 3.812244 | 3.076599 | 3.006201 | 2.931316 |
| TC1100001193.oe.1 | OTTHUMG00000167224 | 0.025358 | 1.5185692 | down | 5.985819 | 5.458585 | 5.770717 | 4.98371 | 5.260696 | 5.162577 |
| TC1100001843.oe.1 | RP11-35J10.6 | 0.010691 | 1.5111418 | down | 3.715259 | 3.658153 | 3.994388 | 3.174078 | 3.343159 | 3.063646 |
| TC1100002612.oe.1 | lnc-STARD10-3 | 0.049209 | 1.6998619 | up | 4.399913 | 4.49966 | 4.361862 | 4.863169 | 4.970057 | 5.724461 |
| TC1100002613.oe.1 | lnc-STARD10-4 | 0.040787 | 1.9247584 | up | 5.829653 | 6.856773 | 6.640076 | 7.298831 | 7.37803 | 7.483672 |
| TC1100002801.oe.1 | RP11-643G5.7 | 0.047572 | 1.5056968 | down | 3.667032 | 3.52413 | 3.094904 | 2.610309 | 2.894058 | 3.010406 |
| TC1200000340.oe.1 | lnc-C12orf39-2 | 0.032759 | 1.5058181 | down | 11.02762 | 11.04994 | 10.86629 | 10.73859 | 10.2498 | 10.18382 |
| TC1200001144.oe.1 | lnc-C12orf63-1 | 0.015036 | 1.5201349 | down | 12.50547 | 12.90902 | 12.49235 | 12.12328 | 12.04197 | 11.92899 |
| TC1200001746.oe.1 | lnc-C12orf4-1 | 0.002573 | 1.5551714 | down | 11.04503 | 11.29769 | 11.10901 | 10.60755 | 10.52292 | 10.41004 |
| TC1300000667.oe.1 | lnc-GPC5-5 | 0.01215 | 1.6162697 | up | 4.522174 | 4.704978 | 4.748758 | 5.311701 | 5.125308 | 5.616905 |
| TC1300001272.oe.1 | lnc-INTS6-6 | 0.020824 | 1.7939171 | up | 3.299518 | 2.805902 | 2.868469 | 3.500938 | 3.991689 | 4.010602 |
| TC1300001431.oe.1 | lnc-EDNRB-9 | 0.019514 | 1.5283378 | down | 3.962384 | 4.171786 | 4.005221 | 3.459694 | 3.164783 | 3.679023 |
| TC1400000507.oe.1 | lnc-PPM1A-3 | 0.011042 | 1.5420059 | up | 3.286383 | 3.40667 | 2.978206 | 3.747887 | 3.944432 | 3.853365 |
| TC1400000892.oe.1 | lnc-AK7-1 | 0.004135 | 1.6619251 | down | 6.72181 | 6.868601 | 6.851193 | 6.167825 | 6.222642 | 5.852569 |
| TC1400000911.oe.1 | lnc-C14orf177-4 | 0.035939 | 1.5215558 | down | 3.748886 | 3.141955 | 3.642922 | 2.911781 | 2.99624 | 2.8091 |
| TC1500001512.oe.1 | lnc-SHC4-1 | 0.033191 | 1.6898153 | down | 8.484748 | 8.495468 | 8.781756 | 8.258546 | 7.670909 | 7.561924 |
| TC1600000184.oe.1 | RP11-295D4.4 | 0.040805 | 1.579507 | up | 6.404532 | 6.117527 | 6.423731 | 7.019385 | 7.293254 | 6.611573 |
| TC1600001476.oe.1 | lnc-GNG13-2 | 0.010886 | 1.5158337 | down | 7.37609 | 6.995629 | 7.152038 | 6.4396 | 6.699541 | 6.584283 |
| TC1600001664.oe.1 | lnc-USP7-1 | 0.019133 | 1.607708 | down | 4.155842 | 4.721453 | 4.493496 | 3.833138 | 3.623907 | 3.858729 |
| TC1600001953.oe.1 | lnc-SEZ6L2-2 | 0.010964 | 1.5349983 | up | 6.965396 | 6.924273 | 7.343631 | 7.627072 | 7.723211 | 7.737728 |
| TC1600002424.oe.1 | lnc-PMFBP1-2 | 0.014606 | 1.5519 | down | 7.766811 | 8.11164 | 7.939092 | 7.523668 | 7.1218 | 7.269967 |
| TC1600002432.oe.1 | lnc-ZFHX3-2 | 0.021977 | 1.6530671 | down | 3.743553 | 3.652024 | 3.291891 | 2.609623 | 3.103511 | 2.798898 |
| TC1600002508.oe.1 | lnc-MAF-6 | 0.007388 | 1.6009406 | down | 3.85557 | 3.543676 | 3.643407 | 2.891341 | 2.914684 | 3.199869 |
| TC1700000477.oe.1 | lnc-TMEM97-2 | 0.010265 | 1.523373 | down | 10.91037 | 10.91488 | 10.7436 | 10.4253 | 10.30282 | 10.01892 |
| TC1800000362.oe.1 | lnc-FHOD3-1 | 0.037699 | 1.5246061 | up | 12.42909 | 12.15038 | 12.65232 | 12.75442 | 13.09717 | 13.20551 |
| TC1800000391.oe.1 | RP11-687E1.2 | 0.019714 | 1.5164605 | down | 7.773538 | 8.250601 | 7.771526 | 7.318146 | 7.348456 | 7.32694 |
| TC1900000742.oe.1 | lnc-ZNF30-4 | 0.040551 | 1.6800605 | down | 4.83023 | 4.34821 | 4.241926 | 4.071964 | 3.542852 | 3.560011 |
| TC1900001903.oe.1 | CTD-3222D19.4 | 1.86E-04 | 1.5297903 | up | 3.704735 | 3.727911 | 3.664834 | 4.228049 | 4.348894 | 4.360538 |
| TC1900002781.oe.1 | lnc-ZNF552-2 | 0.029411 | 1.6787137 | down | 3.808479 | 4.269144 | 4.209088 | 3.51535 | 3.526297 | 3.002995 |
| TC2000000268.oe.1 | lnc-GINS1-6 | 0.004226 | 1.7701757 | up | 4.644072 | 4.633773 | 5.04997 | 5.556551 | 5.660192 | 5.58275 |
| TC2000000281.oe.1 | lnc-FRG1B-1 | 0.002322 | 1.5418694 | down | 7.265215 | 7.476424 | 7.531686 | 6.874036 | 6.735806 | 6.789442 |
| TC2000001145.oe.1 | RP11-425M5.7 | 0.024199 | 1.5621859 | down | 9.42881 | 9.520601 | 9.311004 | 9.010624 | 8.877511 | 8.441582 |
| TSUnmapped00001304.oe.1 | lnc-AL358813.2-2 | 0.013597 | 1.838142 | down | 5.123907 | 5.067049 | 5.603924 | 4.20893 | 4.616769 | 4.334436 |
| TSUnmapped00001693.oe.1 | lnc-APOD-3 | 0.010418 | 1.6750814 | down | 11.44431 | 11.52198 | 11.09415 | 10.80172 | 10.53613 | 10.4899 |
| TC01000012.hg.4 | RP5-857K21.2 | 0.044695 | 1.5910615 | up | 3.067108 | 3.468864 | 3.296468 | 3.713273 | 3.782065 | 4.34707 |
| TC01000070.hg.4 | TPRG1L | 0.007428 | 1.615866 | up | 7.22377 | 7.399243 | 7.481034 | 7.952332 | 7.937628 | 8.291009 |
| TC01000234.hg.4 | RP1-20B21.4 | 0.017173 | 1.5795974 | up | 4.701169 | 4.132277 | 4.339947 | 5.059323 | 5.004232 | 5.088509 |
| TC01000499.hg.4 | RP1-144F13.3 | 0.035727 | 1.6252078 | up | 3.214057 | 3.491901 | 3.901303 | 4.194079 | 4.081499 | 4.433556 |
| TC01000702.hg.4 | NFIA | 0.047655 | 1.6804931 | down | 6.24712 | 6.442472 | 5.757465 | 5.61914 | 5.51543 | 5.065833 |
| TC01000738.hg.4 | MIER1 | 0.001901 | 1.538897 | down | 11.32297 | 11.30435 | 11.08812 | 10.64187 | 10.67049 | 10.53739 |
| TC01000838.hg.4 | RP11-413E1.4 | 0.002999 | 1.5100642 | down | 4.411293 | 4.706112 | 4.664006 | 3.985477 | 3.999353 | 4.012752 |
| TC01000965.hg.4 | LAMTOR5-AS1 | 0.022315 | 1.5508225 | up | 3.067928 | 3.463719 | 3.549062 | 4.054727 | 3.811228 | 4.113854 |
| TC01001302.hg.4 | POU5F1P4 | 0.021535 | 1.6084445 | up | 3.12767 | 2.625386 | 3.017668 | 3.430073 | 3.592224 | 3.805426 |
| TC01001382.hg.4 | FCER1G | 0.005619 | 1.6260684 | down | 3.601129 | 3.316973 | 3.68836 | 2.711746 | 2.859104 | 2.931449 |
| TC01001669.hg.4 | RNPEP | 0.030501 | 1.5260237 | up | 10.32384 | 10.44141 | 10.82174 | 10.94902 | 11.13904 | 11.32826 |
| TC01001781.hg.4 | FLVCR1 | 0.015015 | 1.5369716 | down | 8.869467 | 8.918247 | 8.886867 | 8.545803 | 8.243788 | 8.024718 |
| TC01001819.hg.4 | 2-Mar | 0.048 | 1.551522 | up | 4.942827 | 4.631763 | 5.151481 | 5.622881 | 5.782484 | 5.22176 |
| TC01002379.hg.4 | RP1-317E23.3 | 0.032389 | 1.5576519 | down | 3.843752 | 3.873826 | 3.555586 | 2.897195 | 3.454732 | 3.003119 |
| TC01002416.hg.4 | EYA3 | 0.013869 | 1.5549406 | up | 7.617204 | 7.649314 | 7.602247 | 8.535246 | 8.012484 | 8.231612 |
| TC01002688.hg.4 | RP5-866L20.1 | 0.015121 | 1.6165459 | up | 4.521083 | 4.440017 | 4.593879 | 5.389919 | 4.883705 | 5.360098 |
| TC01002710.hg.4 | RP4-794H19.1 | 0.007812 | 1.6376952 | up | 3.028693 | 2.621022 | 2.85071 | 3.651553 | 3.382512 | 3.60136 |
| TC01002739.hg.4 | RP11-24J23.2 | 0.00105 | 1.6967074 | down | 6.074546 | 6.281614 | 6.087559 | 5.327837 | 5.504596 | 5.32307 |
| TC01002835.hg.4 | RP4-651E10.4 | 0.016843 | 1.8082794 | down | 4.094872 | 3.527743 | 3.427286 | 2.734015 | 2.941387 | 2.810646 |
| TC01003058.hg.4 | SRGAP2-AS1 | 0.003532 | 1.5601186 | up | 3.377924 | 3.260449 | 3.340228 | 4.130807 | 3.79112 | 3.981641 |
| TC01003372.hg.4 | OR10T2 | 0.032197 | 1.8146609 | up | 2.256559 | 2.736305 | 2.740903 | 3.166609 | 3.28863 | 3.857627 |
| TC01003547.hg.4 | RP5-1114G22.2 | 0.013549 | 1.6223141 | down | 4.314387 | 4.189826 | 3.814456 | 3.546218 | 3.321129 | 3.357163 |
| TC01003804.hg.4 | RP11-384C4.7 | 0.003665 | 1.655404 | down | 5.214944 | 5.130028 | 4.998435 | 4.214021 | 4.382893 | 4.564944 |
| TC01006308.hg.4 | FCGR3B | 0.01897 | 1.5527937 | up | 2.968626 | 2.853641 | 2.774265 | 3.203698 | 3.560317 | 3.737116 |
| TC02000049.hg.4 | RP11-400L8.2 | 6.96E-04 | 1.6962111 | down | 3.741561 | 3.647887 | 3.584574 | 2.943384 | 2.764486 | 2.979205 |
| TC02000062.hg.4 | AC092687.5 | 0.032313 | 1.7476354 | down | 4.942318 | 4.407532 | 5.093551 | 4.07837 | 4.207417 | 3.741401 |
| TC02000435.hg.4 | SPR | 0.010833 | 1.5016452 | up | 8.50468 | 8.570244 | 8.642911 | 9.406699 | 9.019307 | 9.051461 |
| TC02000544.hg.4 | AC104134.2 | 0.001553 | 1.5072163 | down | 2.822268 | 2.811354 | 3.012483 | 2.337044 | 2.208189 | 2.325213 |
| TC02000565.hg.4 | ACTR3BP2 | 0.002717 | 1.6629226 | down | 4.272496 | 4.120265 | 4.357824 | 3.554211 | 3.351224 | 3.643988 |
| TC02000662.hg.4 | SULT1C3 | 0.003996 | 1.5319769 | down | 3.593748 | 3.587343 | 3.579779 | 2.977377 | 3.147502 | 2.789807 |
| TC02001273.hg.4 | PKI55 | 0.035489 | 1.5486095 | down | 6.591898 | 6.993045 | 6.404188 | 6.224034 | 6.003094 | 5.869085 |
| TC02001318.hg.4 | FAM134A | 0.024172 | 1.5242676 | up | 5.773477 | 5.316615 | 5.785316 | 6.345528 | 6.266655 | 6.087574 |
| TC02001880.hg.4 | AC007131.2 | 0.043057 | 1.5724428 | down | 2.818303 | 3.037017 | 3.372736 | 2.726089 | 2.323526 | 2.219418 |
| TC02001894.hg.4 | FAM161A | 0.036572 | 1.5970329 | down | 9.51464 | 9.564589 | 9.273136 | 9.02718 | 8.916853 | 8.382148 |
| TC02001961.hg.4 | FAM136A | 0.029757 | 1.5183885 | down | 10.03741 | 10.21805 | 10.25905 | 9.792306 | 9.23748 | 9.6771 |
| TC02002049.hg.4 | ST3GAL5 | 0.049344 | 1.6516129 | down | 5.779005 | 6.056397 | 5.741546 | 4.862822 | 5.613091 | 4.929408 |
| TC02002127.hg.4 | MGAT4A | 0.028891 | 1.6022476 | up | 2.846077 | 2.68759 | 2.547463 | 3.041823 | 3.399946 | 3.679651 |
| TC02002236.hg.4 | AC010982.2 | 0.011181 | 1.8493524 | up | 2.867178 | 2.867055 | 2.975259 | 3.628458 | 4.17972 | 3.562374 |
| TC02002568.hg.4 | TTC30B | 0.013819 | 1.541931 | down | 5.250142 | 5.272176 | 5.161506 | 4.781796 | 4.315469 | 4.712345 |
| TC02002577.hg.4 | SESTD1 | 0.014394 | 1.548065 | down | 7.802947 | 7.661999 | 7.912839 | 6.902066 | 7.347744 | 7.236577 |
| TC02002582.hg.4 | CWC22 | 0.006046 | 1.557934 | down | 15.32203 | 15.32958 | 15.06976 | 14.69418 | 14.67713 | 14.43116 |
| TC02004944.hg.4 | IGKV2-24 | 4.88E-04 | 1.5087817 | up | 2.86894 | 2.941696 | 2.913341 | 3.402978 | 3.515725 | 3.585426 |
| TC02004967.hg.4 | APLF | 0.021431 | 1.5745698 | down | 9.331653 | 9.459811 | 9.333037 | 9.057997 | 8.483768 | 8.61786 |
| TC03000069.hg.4 | PPARG | 0.032977 | 1.5355915 | down | 5.421184 | 5.723639 | 5.969852 | 5.202102 | 4.864589 | 5.1916 |
| TC03000104.hg.4 | MIR3714 | 0.036982 | 1.8300068 | up | 5.518593 | 5.643815 | 6.410302 | 6.646514 | 6.721958 | 6.819785 |
| TC03000416.hg.4 | CTD-2013N24.2 | 0.019677 | 1.8040663 | down | 4.959797 | 4.563529 | 4.536154 | 4.001694 | 4.028257 | 3.475772 |
| TC03000474.hg.4 | ARL13B | 0.039198 | 1.797476 | down | 7.965343 | 7.999129 | 8.060332 | 7.708204 | 6.790766 | 6.987915 |
| TC03000819.hg.4 | CLRN1-AS1 | 0.007819 | 1.8450115 | up | 2.985987 | 2.817001 | 3.029313 | 4.047147 | 3.500514 | 3.93553 |
| TC03000909.hg.4 | SEC62 | 0.021548 | 1.862536 | down | 10.27543 | 9.883408 | 9.566699 | 9.178841 | 9.109169 | 8.745722 |
| TC03000932.hg.4 | NLGN1 | 0.012983 | 1.8570849 | down | 5.50515 | 5.659901 | 5.398463 | 5.01821 | 4.428957 | 4.437227 |
| TC03001041.hg.4 | TPRG1 | 0.03258 | 1.7037762 | down | 4.04705 | 3.554464 | 3.23455 | 2.769166 | 2.860901 | 2.899789 |
| TC03001046.hg.4 | CLDN16 | 0.02091 | 1.5722762 | down | 4.499037 | 4.199116 | 4.73266 | 3.847717 | 3.665109 | 3.959423 |
| TC03001272.hg.4 | CNOT10-AS1 | 0.018051 | 1.5441209 | up | 2.943131 | 3.251307 | 3.067504 | 3.944009 | 3.722286 | 3.476004 |
| TC03001346.hg.4 | TMEM158 | 0.001309 | 1.635101 | down | 5.396077 | 5.236486 | 5.515555 | 4.741622 | 4.620687 | 4.65767 |
| TC03001591.hg.4 | STX19 | 0.040794 | 1.5645391 | up | 2.866747 | 3.183917 | 3.406172 | 4.088169 | 3.718936 | 3.586944 |
| TC03001913.hg.4 | RP11-362A9.3 | 0.007022 | 1.5550662 | down | 2.962936 | 3.120943 | 3.112925 | 2.506585 | 2.575299 | 2.203992 |
| TC03001948.hg.4 | LXN | 0.016107 | 1.7630857 | down | 4.158564 | 4.010515 | 4.342148 | 3.442728 | 3.609836 | 3.004354 |
| TC03002043.hg.4 | RP11-496B10.3 | 0.029116 | 1.5562637 | up | 3.008368 | 2.716479 | 2.35507 | 3.392699 | 3.284768 | 3.31671 |
| TC04000181.hg.4 | RBPJ | 0.012011 | 1.6895717 | down | 9.544737 | 9.70267 | 9.642289 | 9.130318 | 8.929671 | 8.559732 |
| TC04000319.hg.4 | SRD5A3 | 0.02332 | 1.5478649 | down | 9.425212 | 9.80045 | 9.248751 | 8.815218 | 8.772836 | 8.99552 |
| TC04000554.hg.4 | SGMS2 | 0.027185 | 1.6550046 | up | 3.452098 | 2.947181 | 3.173664 | 3.733269 | 4.227576 | 3.792604 |
| TC04000583.hg.4 | LARP7 | 0.009096 | 1.5344803 | down | 14.12255 | 14.07895 | 13.80018 | 13.54134 | 13.26224 | 13.34485 |
| TC04000653.hg.4 | C4orf33 | 0.041507 | 1.5219976 | down | 7.458505 | 7.957205 | 7.405009 | 7.136659 | 6.794992 | 7.07117 |
| TC04000662.hg.4 | RP11-149A7.2 | 0.00188 | 1.8796273 | up | 3.473286 | 3.582121 | 3.319218 | 4.246961 | 4.56442 | 4.294583 |
| TC04000947.hg.4 | ZNF732 | 0.031878 | 1.593676 | down | 3.138947 | 2.785904 | 3.493258 | 2.402321 | 2.460531 | 2.538182 |
| TC04001056.hg.4 | RP11-484O2.1 | 0.007703 | 1.6374903 | down | 5.08351 | 4.650543 | 4.77401 | 4.141369 | 4.007713 | 4.224522 |
| TC04001460.hg.4 | RPL34-AS1 | 0.0231 | 1.5270611 | up | 3.411849 | 3.515158 | 3.120708 | 3.803451 | 3.87414 | 4.202396 |
| TC04001482.hg.4 | RP11-119H12.6 | 0.040842 | 1.8973687 | up | 3.249142 | 2.656688 | 3.135017 | 3.719615 | 3.653583 | 4.439649 |
| TC04001621.hg.4 | SLC10A7 | 0.001805 | 1.5496345 | down | 8.091988 | 8.120333 | 8.149453 | 7.530802 | 7.608634 | 7.326554 |
| TC04001718.hg.4 | DDX60 | 0.034497 | 1.8583018 | down | 5.928558 | 5.114262 | 5.237412 | 4.498178 | 4.330947 | 4.769154 |
| TC04001723.hg.4 | CBR4 | 0.048541 | 1.7540802 | down | 7.888572 | 7.83039 | 8.375498 | 7.658048 | 7.134464 | 6.869804 |
| TC04001748.hg.4 | LOC101928509 | 0.017839 | 1.624139 | up | 3.16843 | 3.110348 | 3.669465 | 4.063093 | 4.029018 | 3.955157 |
| TC04002914.hg.4 | CYP4V2 | 0.011911 | 1.7526442 | down | 9.770802 | 9.986676 | 9.572058 | 9.237926 | 8.899341 | 8.763668 |
| TC05000002.hg.4 | PLEKHG4B | 0.020278 | 1.5521905 | up | 3.399051 | 3.42007 | 3.799981 | 4.010229 | 4.130719 | 4.381071 |
| TC05000259.hg.4 | AC104113.3 | 0.007129 | 1.5911114 | down | 3.210668 | 3.407427 | 3.582358 | 2.720365 | 2.601791 | 2.868193 |
| TC05000291.hg.4 | PIK3R1 | 0.026654 | 1.5679578 | down | 12.01849 | 12.19931 | 12.02184 | 11.4476 | 11.73376 | 11.11162 |
| TC05000629.hg.4 | CSF2 | 0.005343 | 1.5202078 | up | 4.921141 | 4.935929 | 4.648696 | 5.507509 | 5.324031 | 5.487031 |
| TC05000635.hg.4 | SLC22A5 | 0.033833 | 1.5276922 | up | 5.83138 | 5.816095 | 6.230374 | 6.801855 | 6.327541 | 6.582512 |
| TC05000886.hg.4 | UBLCP1 | 0.046777 | 1.5732163 | down | 17.06866 | 17.04696 | 16.70905 | 16.18978 | 16.66983 | 16.00391 |
| TC05000971.hg.4 | CTC-430J12.2 | 0.049589 | 1.5822594 | up | 2.359768 | 2.758377 | 2.417793 | 3.54865 | 3.120378 | 2.852868 |
| TC05001069.hg.4 | BTNL3 | 0.049111 | 1.5563241 | up | 4.202074 | 3.805424 | 3.583982 | 4.230686 | 4.694463 | 4.580759 |
| TC05001493.hg.4 | CTC-366B18.2 | 0.025286 | 1.558394 | up | 2.937503 | 2.832544 | 2.777263 | 3.748714 | 3.149223 | 3.569553 |
| TC05001688.hg.4 | CCDC112 | 0.022283 | 1.5224952 | down | 12.73698 | 13.13423 | 12.70041 | 12.43118 | 12.202 | 12.11913 |
| TC06000061.hg.4 | SNRNP48 | 0.014911 | 1.5287579 | down | 11.17066 | 11.33823 | 10.90607 | 10.68734 | 10.43697 | 10.45357 |
| TC06000334.hg.4 | TRIM15 | 0.022353 | 1.6443805 | up | 2.583541 | 3.177504 | 2.899138 | 3.62983 | 3.761944 | 3.421041 |
| TC06000383.hg.4 | MSH5-SAPCD1 | 0.004005 | 1.747461 | up | 5.917693 | 5.572254 | 5.506051 | 6.395007 | 6.550994 | 6.465778 |
| TC06000740.hg.4 | RP11-554D15.4 | 0.014784 | 1.7404294 | up | 3.219404 | 3.144804 | 2.64994 | 3.916087 | 3.840681 | 3.65571 |
| TC06000821.hg.4 | RP3-417O22.3 | 0.034222 | 1.5928723 | up | 3.122407 | 3.279667 | 2.701319 | 3.798831 | 3.460359 | 3.859094 |
| TC06000952.hg.4 | --- | 0.011813 | 1.8856494 | up | 2.906475 | 2.255244 | 2.363782 | 3.517122 | 3.424133 | 3.32943 |
| TC06001287.hg.4 | RP11-146I2.1 | 0.010816 | 1.5418757 | down | 3.00589 | 3.158465 | 3.078288 | 2.210732 | 2.496594 | 2.661258 |
| TC06001588.hg.4 | BAK1 | 0.033773 | 1.7607054 | up | 5.312599 | 5.168327 | 5.799001 | 6.58392 | 6.117682 | 6.026787 |
| TC06001869.hg.4 | KHDC1L | 0.024673 | 2.102704 | down | 3.193012 | 3.362848 | 4.094934 | 2.736885 | 2.349564 | 2.347607 |
| TC06001984.hg.4 | RTN4IP1 | 0.042012 | 1.5582621 | up | 4.879981 | 5.062439 | 5.3112 | 5.631563 | 6.067487 | 5.474384 |
| TC06002119.hg.4 | VNN1 | 0.011457 | 1.5300282 | down | 8.045225 | 7.682282 | 8.11041 | 7.390662 | 7.346819 | 7.25976 |
| TC06002124.hg.4 | SLC2A12 | 0.022071 | 1.8866063 | down | 4.386763 | 4.390275 | 3.945956 | 3.712538 | 3.244025 | 3.019051 |
| TC06002238.hg.4 | SYNE1 | 0.013588 | 1.8150873 | up | 3.571182 | 2.96225 | 3.508652 | 4.331099 | 4.106544 | 4.184558 |
| TC06004089.hg.4 | RP11-446F17.3 | 0.003968 | 1.58203 | down | 3.760435 | 4.130808 | 3.982259 | 3.299276 | 3.247705 | 3.34119 |
| TC06004098.hg.4 | PHACTR2 | 0.026186 | 1.546518 | down | 9.778888 | 9.949138 | 9.416334 | 9.110194 | 9.233387 | 8.913708 |
| TC07000710.hg.4 | LOC101928012 | 0.003212 | 1.8317821 | up | 2.876453 | 2.815115 | 2.977446 | 4.007288 | 3.564438 | 3.717031 |
| TC07000957.hg.4 | ARHGEF5 | 0.033513 | 1.6924288 | down | 7.747985 | 7.698027 | 8.262124 | 7.456308 | 6.992921 | 6.981622 |
| TC07001318.hg.4 | INHBA | 0.001235 | 1.6431303 | down | 12.49623 | 12.67958 | 12.3779 | 11.80939 | 11.79042 | 11.80456 |
| TC07001366.hg.4 | SUN3 | 0.014684 | 1.9187851 | up | 6.232248 | 6.123706 | 5.900416 | 6.615115 | 7.271592 | 7.190241 |
| TC07001473.hg.4 | RP4-736H5.3 | 0.011376 | 1.7128295 | up | 2.926893 | 3.161042 | 2.936416 | 3.978971 | 3.901735 | 3.47279 |
| TC07001753.hg.4 | C7orf66 | 0.004143 | 1.5379833 | down | 3.185069 | 2.939119 | 3.045853 | 2.293382 | 2.560748 | 2.452792 |
| TC07001764.hg.4 | TMEM168 | 0.046863 | 1.5700694 | down | 9.256618 | 8.901016 | 9.289333 | 8.867688 | 8.406889 | 8.219905 |
| TC07001819.hg.4 | IQUB | 0.038194 | 1.5728064 | up | 3.271046 | 2.825385 | 2.725529 | 3.544278 | 3.846543 | 3.391161 |
| TC07002077.hg.4 | PTPRN2 | 0.014385 | 1.5149169 | up | 3.16295 | 3.152734 | 2.977954 | 3.95395 | 3.619602 | 3.517802 |
| TC07003393.hg.4 | MIR6837 | 0.015158 | 1.7069913 | up | 8.202574 | 8.426663 | 8.794256 | 9.36696 | 9.100066 | 9.270837 |
| TC08000066.hg.4 | RP11-10A14.4 | 0.021363 | 1.8894316 | down | 4.515195 | 4.018423 | 4.740597 | 3.767087 | 3.390655 | 3.362616 |
| TC08000408.hg.4 | RP11-379I19.3 | 0.002014 | 1.9017161 | down | 4.442037 | 4.169523 | 4.366576 | 3.48299 | 3.515347 | 3.197894 |
| TC08000439.hg.4 | TRIM55 | 0.026092 | 1.567803 | down | 3.442439 | 4.031807 | 3.92671 | 3.243602 | 3.07388 | 3.137241 |
| TC08000517.hg.4 | RP11-1149M10.2 | 0.002558 | 1.5509627 | up | 3.23926 | 3.099241 | 3.166173 | 3.64047 | 3.833532 | 3.930163 |
| TC08000542.hg.4 | CNBD1 | 0.009497 | 1.8118534 | up | 2.439547 | 2.069304 | 2.199388 | 2.913176 | 3.387033 | 2.980429 |
| TC08000922.hg.4 | DEFB104B | 0.036871 | 1.743422 | up | 2.410551 | 2.620363 | 2.912219 | 3.581861 | 3.739264 | 3.027774 |
| TC08001196.hg.4 | RP11-567J20.1 | 0.002287 | 2.0547566 | up | 3.100699 | 3.253059 | 3.049964 | 4.268657 | 3.903182 | 4.348785 |
| TC08001717.hg.4 | ZC3H3 | 0.022932 | 1.5476345 | down | 3.144358 | 3.648122 | 3.35035 | 2.611588 | 2.937282 | 2.703766 |
| TC09000362.hg.4 | LOC101927502 | 0.029991 | 1.5699706 | up | 2.802931 | 3.085447 | 2.438472 | 3.32419 | 3.538829 | 3.416043 |
| TC09000539.hg.4 | TMEM38B | 0.014341 | 1.5032125 | down | 11.37393 | 11.30445 | 11.58583 | 11.03069 | 10.83342 | 10.63595 |
| TC09000752.hg.4 | POMT1 | 0.009856 | 1.7834798 | up | 6.921764 | 7.036445 | 7.389275 | 8.16801 | 7.787599 | 7.89596 |
| TC09000860.hg.4 | FAM138C | 0.025996 | 1.5039651 | up | 3.224136 | 3.571783 | 3.443233 | 3.766442 | 3.997865 | 4.241159 |
| TC09000940.hg.4 | SLC24A2 | 0.006107 | 1.6245159 | up | 2.709313 | 2.529241 | 2.561171 | 3.245519 | 3.124693 | 3.529543 |
| TC09001095.hg.4 | ZNF658 | 0.042123 | 1.501605 | down | 7.613904 | 6.972672 | 7.494255 | 6.803789 | 6.714648 | 6.80288 |
| TC09001220.hg.4 | NMRK1 | 0.009194 | 1.6867863 | down | 6.96693 | 6.920807 | 6.590667 | 6.22547 | 6.125079 | 5.865024 |
| TC09001286.hg.4 | SLC28A3 | 0.036859 | 1.7799156 | up | 3.364295 | 3.529414 | 2.867966 | 3.84669 | 4.444285 | 3.966126 |
| TC0X000256.hg.4 | WDR13 | 0.029953 | 1.5212559 | up | 4.83338 | 4.696493 | 5.151604 | 5.349224 | 5.401979 | 5.746061 |
| TC0X000499.hg.4 | TCEAL4 | 0.027281 | 1.8065284 | down | 13.53671 | 13.65926 | 13.26161 | 12.66616 | 12.9988 | 12.23296 |
| TC0X000530.hg.4 | ATG4A | 0.007267 | 1.7314066 | down | 9.694454 | 9.729994 | 9.623273 | 9.174643 | 8.851252 | 8.645997 |
| TC0X001092.hg.4 | ARHGEF9 | 0.007069 | 1.9922084 | down | 10.8642 | 11.28761 | 10.65315 | 9.938291 | 10.04396 | 9.839602 |
| TC0X001182.hg.4 | BRWD3 | 0.014428 | 1.7046549 | down | 9.536513 | 9.778002 | 9.769439 | 8.730214 | 9.260511 | 8.78479 |
| TC0X001315.hg.4 | UPF3B | 0.041182 | 1.6082041 | down | 13.72395 | 13.73428 | 13.10416 | 12.85395 | 12.65453 | 12.99756 |
| TC0X001432.hg.4 | AC004070.1 | 0.00618 | 1.508313 | down | 2.824619 | 3.037912 | 3.143907 | 2.52561 | 2.386504 | 2.315516 |
| TC0Y000070.hg.4 | TTTY9B | 0.011036 | 1.5635406 | down | 3.595364 | 3.681959 | 3.355357 | 2.915714 | 2.7083 | 3.074216 |
| TC0Y000192.hg.4 | LOC100509646 | 0.020072 | 1.784228 | up | 3.159775 | 2.963512 | 2.963006 | 3.437993 | 4.096923 | 4.057277 |
| TC10000194.hg.4 | RP11-218D6.4 | 7.76E-04 | 1.5267985 | down | 3.980279 | 3.979929 | 3.788088 | 3.313286 | 3.27211 | 3.331371 |
| TC10000299.hg.4 | LOC101927699 | 0.01134 | 1.545014 | up | 3.43341 | 3.485914 | 3.593909 | 4.05129 | 3.952451 | 4.392352 |
| TC10000313.hg.4 | FAM25BP | 0.021534 | 1.5856395 | down | 4.794188 | 4.791401 | 4.562076 | 3.729921 | 4.274089 | 4.148461 |
| TC10000460.hg.4 | LOC101929112 | 0.049149 | 1.5253218 | down | 7.205248 | 7.653952 | 7.049039 | 6.468285 | 6.883532 | 6.729079 |
| TC10000706.hg.4 | R3HCC1L | 0.046854 | 1.5876334 | down | 6.864644 | 6.971026 | 7.075256 | 6.563045 | 6.495716 | 5.851531 |
| TC10000761.hg.4 | RNU6-43P | 0.04804 | 1.9552472 | down | 9.724297 | 8.819327 | 8.603601 | 8.058863 | 8.081553 | 8.104757 |
| TC10000766.hg.4 | CYP17A1-AS1 | 0.012406 | 1.7122349 | up | 2.876371 | 2.416863 | 2.889888 | 3.59504 | 3.324459 | 3.591264 |
| TC10000868.hg.4 | BAG3 | 0.029814 | 1.519377 | up | 7.66567 | 7.305278 | 7.647021 | 8.2809 | 7.86258 | 8.284927 |
| TC10001292.hg.4 | A1CF | 0.018319 | 1.9698191 | down | 3.435642 | 3.800212 | 3.065091 | 2.593047 | 2.176038 | 2.597671 |
| TC10001320.hg.4 | RHOBTB1 | 0.04338 | 1.6997015 | down | 3.869931 | 4.149942 | 4.184058 | 2.90195 | 3.740455 | 3.265682 |
| TC10001426.hg.4 | RP11-77G23.5 | 0.004024 | 1.6042151 | up | 6.55914 | 6.425356 | 6.272056 | 6.950003 | 7.133178 | 7.218974 |
| TC10001538.hg.4 | FRA10AC1 | 0.003661 | 1.6574236 | down | 7.479283 | 7.273029 | 7.558603 | 6.842901 | 6.727274 | 6.553914 |
| TC10001659.hg.4 | PDCD4-AS1 | 0.034459 | 1.5129541 | up | 2.642184 | 3.211476 | 3.116565 | 3.521096 | 3.513602 | 3.727632 |
| TC11000542.hg.4 | FADS2 | 0.042653 | 1.5476562 | up | 8.188062 | 8.253238 | 8.807541 | 9.110965 | 8.87488 | 9.15325 |
| TC11000625.hg.4 | TIGD3 | 0.029912 | 1.570659 | down | 4.830543 | 4.675599 | 5.281482 | 4.393461 | 4.307937 | 4.132117 |
| TC11000944.hg.4 | ARHGAP42 | 0.019122 | 1.6418464 | down | 7.403138 | 7.639408 | 7.274623 | 7.029273 | 6.524932 | 6.617006 |
| TC11001030.hg.4 | NXPE2 | 0.005955 | 1.6215234 | down | 3.323434 | 3.594788 | 3.387537 | 2.650952 | 2.621636 | 2.941122 |
| TC11001395.hg.4 | NRIP3 | 0.023507 | 1.8512417 | down | 4.797821 | 5.042436 | 4.627976 | 4.349772 | 3.842976 | 3.610005 |
| TC11002059.hg.4 | PDE2A | 0.003966 | 1.5978708 | down | 3.540419 | 3.495333 | 3.375278 | 2.600709 | 2.834536 | 2.947333 |
| TC11002211.hg.4 | MAML2 | 0.010768 | 1.6689527 | down | 6.861084 | 7.256388 | 6.851167 | 6.431008 | 6.213498 | 6.107304 |
| TC11002250.hg.4 | CARD18 | 0.042517 | 1.5058373 | up | 2.31335 | 2.661989 | 2.821597 | 3.380327 | 2.93154 | 3.256767 |
| TC11002316.hg.4 | CADM1 | 0.002447 | 1.5503823 | down | 4.703335 | 4.424196 | 4.644304 | 3.950631 | 3.896044 | 4.02729 |
| TC11002459.hg.4 | LOC101929637 | 0.02118 | 1.6510757 | up | 3.090934 | 2.948313 | 2.734644 | 3.483473 | 3.478565 | 3.982072 |
| TC12000159.hg.4 | GABARAPL1 | 0.043364 | 1.549801 | down | 10.5418 | 10.56171 | 10.26086 | 9.524147 | 10.18572 | 9.758253 |
| TC12001657.hg.4 | PPM1H | 0.002981 | 1.5073274 | up | 6.102601 | 5.910377 | 6.143265 | 6.678199 | 6.532357 | 6.721665 |
| TC12001689.hg.4 | GRIP1 | 0.038323 | 1.5748551 | down | 5.518486 | 5.422428 | 5.507969 | 4.892838 | 5.16018 | 4.430208 |
| TC13000349.hg.4 | DOCK9-AS1 | 0.010874 | 1.5217592 | up | 3.278139 | 2.882001 | 2.996738 | 3.562994 | 3.784222 | 3.626882 |
| TC13000374.hg.4 | DAOA | 0.048813 | 1.6708633 | down | 3.782461 | 3.234514 | 3.236848 | 2.294038 | 2.874361 | 2.863644 |
| TC13000449.hg.4 | ANKRD26P3 | 0.043489 | 1.6012304 | down | 4.046731 | 4.066397 | 3.904547 | 3.544865 | 3.563236 | 2.872032 |
| TC13000614.hg.4 | CCDC122 | 0.04212 | 1.6528431 | down | 8.518439 | 8.870171 | 9.164762 | 7.81567 | 8.213592 | 8.349258 |
| TC13000660.hg.4 | CAB39L | 0.028589 | 1.5806069 | down | 7.370555 | 7.129767 | 7.328332 | 6.980734 | 6.418415 | 6.448071 |
| TC13000883.hg.4 | ARHGEF7-AS1 | 0.008536 | 1.52444 | down | 3.897959 | 4.033825 | 4.161894 | 3.45342 | 3.580052 | 3.235367 |
| TC13001725.hg.4 | CCDC169 | 0.031791 | 1.5342578 | down | 6.718492 | 7.16222 | 6.82772 | 6.451539 | 6.014891 | 6.389379 |
| TC14000967.hg.4 | EMC9 | 0.011098 | 1.506288 | up | 8.426407 | 8.698567 | 8.753169 | 9.2019 | 9.077324 | 9.371913 |
| TC15000881.hg.4 | SLCO3A1 | 0.005319 | 1.6247848 | up | 5.920268 | 6.052696 | 6.257295 | 6.890058 | 6.619647 | 6.821301 |
| TC15001337.hg.4 | CEP152 | 0.013905 | 1.642038 | down | 10.9206 | 10.69749 | 10.94497 | 10.2014 | 10.36511 | 9.850089 |
| TC15002810.hg.4 | LOC101930343 | 0.033568 | 1.5469478 | up | 4.182789 | 4.091676 | 4.153841 | 4.381313 | 4.996788 | 4.938479 |
| TC16000126.hg.4 | DNASE1 | 0.030449 | 1.5662669 | up | 3.978579 | 3.702698 | 3.823987 | 4.252361 | 4.83808 | 4.356813 |
| TC16000798.hg.4 | HCFC1R1 | 0.002048 | 1.6708593 | down | 3.894706 | 4.163465 | 4.035964 | 3.369652 | 3.15313 | 3.349582 |
| TC16000831.hg.4 | UBALD1 | 0.041355 | 1.5132982 | down | 9.741498 | 9.629269 | 9.208521 | 9.061601 | 9.034727 | 8.689871 |
| TC16002067.hg.4 | HN1L | 0.046614 | 1.5418224 | up | 10.0157 | 9.63633 | 10.16647 | 10.81151 | 10.28532 | 10.59558 |
| TC16002091.hg.4 | TMEM231 | 0.002894 | 1.5205094 | up | 5.432438 | 5.370481 | 5.335026 | 5.883453 | 5.907677 | 6.160478 |
| TC17000643.hg.4 | RP11-1079K10.2 | 0.038243 | 2.3620641 | down | 3.802105 | 5.027719 | 4.366472 | 3.559464 | 2.926392 | 2.990296 |
| TC17001131.hg.4 | MYH8 | 0.001727 | 1.570031 | up | 2.609893 | 2.673086 | 2.748594 | 3.340878 | 3.45531 | 3.187764 |
| TC17001780.hg.4 | AC015923.1 | 0.021783 | 1.5130582 | down | 4.573134 | 4.165136 | 4.10667 | 3.736849 | 3.775111 | 3.540578 |
| TC18000524.hg.4 | CCDC68 | 0.037785 | 1.6681751 | down | 9.582501 | 9.534655 | 9.42418 | 9.123913 | 8.879488 | 8.323123 |
| TC18000564.hg.4 | DSEL | 0.034642 | 1.5602168 | down | 8.461651 | 8.613715 | 8.849667 | 8.321252 | 7.934986 | 7.743557 |
| TC19000212.hg.4 | CTD-2006C1.2 | 0.028334 | 1.5562187 | down | 5.321861 | 5.141681 | 5.721499 | 4.898242 | 4.614078 | 4.758587 |
| TC19000384.hg.4 | ZNF486 | 0.0325 | 1.5062883 | down | 4.199429 | 3.687864 | 4.117448 | 3.375228 | 3.586621 | 3.269898 |
| TC19000393.hg.4 | ZNF429 | 0.035497 | 1.5181818 | down | 9.95298 | 10.30728 | 10.01609 | 9.684032 | 9.611123 | 9.174161 |
| TC19000756.hg.4 | NR1H2 | 0.011988 | 1.7162035 | up | 4.949825 | 4.933788 | 5.447182 | 5.915775 | 5.777538 | 5.975144 |
| TC19001187.hg.4 | ELOF1 | 0.030788 | 1.8407769 | up | 6.248869 | 6.10121 | 6.50375 | 7.611593 | 6.779379 | 7.1038 |
| TC19001289.hg.4 | ISYNA1 | 0.008484 | 1.6904151 | up | 8.01618 | 8.040066 | 8.378897 | 9.096495 | 8.738506 | 8.872274 |
| TC19001438.hg.4 | ZNF792 | 0.027442 | 1.5481821 | down | 7.681648 | 7.47049 | 7.789392 | 7.035898 | 6.72948 | 7.284426 |
| TC19001623.hg.4 | RTN2 | 8.16E-04 | 1.5465424 | down | 5.039547 | 5.075682 | 4.90344 | 4.287029 | 4.429575 | 4.414927 |
| TC19001846.hg.4 | AC008746.12 | 9.96E-04 | 1.6054196 | down | 6.793955 | 6.954522 | 6.991128 | 6.312597 | 6.241529 | 6.136629 |
| TC19002622.hg.4 | QTRT1 | 0.001462 | 1.5590137 | up | 4.534637 | 4.567378 | 4.604449 | 5.368643 | 5.136428 | 5.123293 |
| TC19002722.hg.4 | RDH13 | 0.020711 | 1.8163335 | up | 4.898944 | 5.392998 | 5.076814 | 6.194705 | 5.621797 | 6.135344 |
| TC20000096.hg.4 | MACROD2 | 0.005185 | 1.5259113 | down | 5.173259 | 5.233551 | 5.016687 | 4.61235 | 4.62846 | 4.353672 |
| TC20000756.hg.4 | BCL2L1 | 0.048009 | 1.5272671 | up | 11.87491 | 11.93892 | 12.45501 | 12.67659 | 12.51358 | 12.91153 |
| TC20000891.hg.4 | SPATA25 | 0.045334 | 1.505331 | up | 3.997716 | 4.342622 | 4.525307 | 5.113156 | 4.877656 | 4.645074 |
| TC20000999.hg.4 | SYCP2 | 0.036969 | 2.3952618 | down | 5.542962 | 5.069106 | 5.275702 | 4.697476 | 4.047927 | 3.361816 |
| TC20001008.hg.4 | LAMA5 | 0.049184 | 1.5051901 | up | 7.828975 | 7.946167 | 8.067649 | 8.828483 | 8.155087 | 8.629058 |
| TC20001761.hg.4 | NFS1 | 0.049619 | 1.5607388 | up | 8.945521 | 8.727553 | 9.483094 | 9.79841 | 9.624846 | 9.659599 |
| TC21000034.hg.4 | C21ORF116 | 0.048266 | 1.8486536 | up | 2.909546 | 2.541844 | 3.312898 | 3.671487 | 4.244424 | 3.507802 |
| TC21000229.hg.4 | C21orf90 | 0.033127 | 1.5239114 | up | 3.98433 | 3.574532 | 3.433409 | 4.288126 | 4.426888 | 4.100593 |
| TC21000254.hg.4 | FTCD-AS1 | 0.008937 | 1.5812358 | up | 3.268049 | 3.635358 | 3.35281 | 3.940519 | 4.229693 | 4.069162 |
| TC21000411.hg.4 | DNAJC28 | 0.03963 | 1.6424863 | down | 7.086752 | 6.835706 | 6.623158 | 5.788184 | 6.140342 | 6.469445 |
| TC21000547.hg.4 | COL18A1-AS2 | 0.038286 | 1.6274924 | up | 3.284149 | 3.616796 | 3.467112 | 4.137538 | 4.532352 | 3.806121 |
| TC22000888.hg.4 | GTSE1-AS1 | 0.007051 | 1.5243597 | up | 3.113174 | 3.119496 | 3.317698 | 3.839572 | 3.601414 | 3.933992 |
| TC14000645.hg.4 | MEG3 | 0.002162 | 2.5195331 | down | 6.111794 | 6.931066 | 7.033623 | 4.583007 | 4.338507 | 4.666278 |

Table S2. Primers used for qRT-PCR

| Gene Symbol | Primer sequence(5'-3') | Products |
| --- | --- | --- |
| h ACTB-F1 | GGCACTCTTCCAGCCTTCC | 255bp |
| h ACTB-R1 | GAGCCGCCGATCCACAC |  |
| MEG3-F1 | CCTGCTGCCCATCTACACCTC | 286bp |
| MEG3-R1 | CCTCTTCATCCTTTGCCATCCTGG |  |
| h p53-F2 | GACGGAGGTTGTGAGGCG | 265bp |
| h p53-R2 | CAGTGTGATGATGGTGAGGATG |  |
| hHif-1α-F | GAGGAAATGAGAGAAATGCTTACA | 206bp |
| hHif-1α-R | CACTGAGGTTGGTTACTGTTGG |  |
| h ICAM1-F1 | TGTGTCCCCCTCAAAAGTCA | 268bp |
| h ICAM1-R1 | TGCCAGTTCCACCCGTTC |  |
| hVEGF-F1 | TCGCTTACTCTCACCTGCTTCT | 239 bp |
| hVEGF-R1 | CAACCACTCACACACACACAAC |  |
| hCRP-F1 | TTTCTTCATCCCCGCATTC | 192bp |
| hCRP-R1 | TGTCTCACTCCCAAAGTCCATA |  |

Table S3. Antibodies used for western blotting

| Antibody Symbol | Dilution ratio | Source |
| --- | --- | --- |
| p53 | 1：200 | Santa |
| p-p53 | 1：1000 | CST |
| CRP | 1：500 | ABCAM |
| HIF-1α | 1：1000 | CST |
| ICAM-1 | 1：1000 | ABCAM |
